# Space‐Confined Synthesis of Thinner Ether‐Functionalized Nanofiltration Membranes with Coffee Ring Structure for Li^+^/Mg^2+^ Separation

**DOI:** 10.1002/advs.202404150

**Published:** 2024-09-13

**Authors:** Wentong Meng, Sifan Chen, Pu Chen, Feng Gao, Jianguo Lu, Yang Hou, Qinggang He, Xiaoli Zhan, Qinghua Zhang

**Affiliations:** ^1^ College of Chemical and Biological Engineering Zhejiang University Hangzhou 310027 China; ^2^ School of Materials Science and Engineering Zhejiang University Hangzhou 310027 China

**Keywords:** li+/mg2+ separation, lithium resource extraction, oligoether modification, space‐confined synthesis

## Abstract

Positively charged nanofiltration membranes have attracted much attention in the field of lithium extraction from salt lakes due to their excellent ability to separate mono‐ and multi‐valent cations. However, the thicker selective layer and the lower affinity for Li^+^ result in lower separation efficiency of the membranes. Here, PEI‐P membranes with highly efficient Li^+^/Mg^2+^ separation performance are prepared by introducing highly lithophilic 4,7,10‐Trioxygen‐1,13‐tridecanediamine (DCA) on the surface of PEI‐TMC membranes using a post‐modification method. Characterization and experimental results show that the utilization of the DCA‐TMC crosslinked structure as a space‐confined layer to inhibit the diffusion of the monomer not only increases the positive charge density of the membrane but also reduces its thickness by ≈35% and presents a unique coffee‐ring structure, which ensures excellent water permeability and rejection of Mg^2+^. The ion–dipole interaction of the ether chains with Li^+^ facilitates Li^+^ transport and improves the Li^+^/Mg^2+^ selectivity (*S_Li,Mg_* = 23.3). In a three‐stage nanofiltration process for treating simulated salt lake water, the PEI‐P membrane can reduce the Mg^2+^/Li^+^ ratio of the salt lake by 400‐fold and produce Li_2_CO_3_ with a purity of more than 99.5%, demonstrating its potential application in lithium extraction from salt lakes.

## Graphical abstract

The physical structure of the membrane is improved by the introduction the space‐confined layer at the top of the membrane to inhibit the upward diffusion of polyethyleneimine. The membrane exhibits excellent Li^+^/Mg^2+^ selectivity as well as Li^+^ permeability by exploiting the high affinity of the ether chain for Li^+^ and shows high potential in the extraction of high purity lithium.

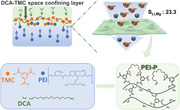

## 1 Introduction

Lithium has received widespread interest for its application in the field of new energy materials due to its extremely high electrochemical activity, specific heat capacity, and redox potential.^[^ ^1^ ^]^ In recent years, with the rapid development of new energy vehicles and energy storage technology, the market demand for lithium resources is expected to grow sharply at a rate of 20% per year.^[^ ^2^ ^]^ Lithium resources are mainly found in solid lithium minerals as well as in salt lake brines in liquid form. The lithium extraction process from ores typically suffers from environmental pollution and high energy consumption, whereas lithium recovery from brines can reduce costs by 30–50%.^[^ ^3^ ^]^ However, the high concentration of coexisting Mg^2+^ in brines, whose hydrated ionic size is similar to that of Li^+^, hinders the acquisition of high‐purity lithium resources. Researchers have attempted to extract lithium from salt lake brines using solvent extraction,^[^ ^4^ ^]^ adsorption,^[^ ^5^ ^]^ and electrochemical methods.^[^ ^6^ ^]^ Nevertheless, the high costs,^[^ ^7^ ^]^ limited adsorption capacity, and cycling ability of adsorbents, as well as environmental concerns,^[^ ^8^ ^]^ still need to be further addressed to improve lithium extraction efficiency.

Membrane separation technology has emerged as one of the ideal choices for obtaining lithium resources due to its high energy efficiency and high selectivity. Based on the size sieving effect and Donnan exclusion effect, nanofiltration (NF) membranes can reject multivalent ions while exhibiting better permeability for monovalent ions, thereby achieving lithium–magnesium separation. The most classic NF membranes are polyamide membranes formed by interfacial polymerization (IP) of piperazine (PIP) and trimesoyl chloride (TMC). However, these membranes are inherently negatively charged, exhibiting poor rejection for Mg^2+^, making it difficult to achieve a high Li^+^/Mg^2+^ separation ratio.^[^ ^9^ ^]^ According to Li's research,^[^ ^10^ ^]^ positively charged NF membranes exhibit stronger repulsion toward cations with higher positive charges, enabling more effective separation of Li^+^ and Mg^2+^. Therefore, positively charged PEI‐TMC membranes formed by the reaction between polyethyleneimine (PEI) as the aqueous monomer and TMC have been widely used in studying Li^+^/Mg^2+^ separation. For instance, Xu et al. prepared composite NF membranes via interfacial polymerization of PEI and TMC, exhibiting a high rejection for Mg^2+^ (95%), but a relatively low pure water permeability (5.0 L m^−2^ h^−1^ bar^−1^).^[^ ^11^ ^]^ Li et al. in situ mixed PEI with crown ether monomers as the aqueous phase to fabricate PEI@15C5‐TMC NF membranes.^[^ ^12^ ^]^ The obtained membranes achieved a Li^+^/Mg^2+^ separation factor of 14 and a permeance of 8.0 L m^−2^ h^−1^ bar^−1^. Wu et al. also introduced 3‐diamino‐methyl‐cyclohexyl triethoxysilane into PEI‐based NF membranes, enhancing their permeability and selectivity.^[^ ^13^ ^]^ However, in situ blending of functional materials with PEI monomers poses challenges. On one hand, the relatively large side‐chain structure of PEI itself cannot avoid the formation of a thicker separation layer in the composite membrane, affecting its permeability.^[^ ^14^ ^]^ On the other hand, after mixing the functional materials with PEI, the in situ interfacial polymerization of the PEI‐TMC membrane results in a higher crosslinking density, which will increase the energy barrier for lithium ions to cross the membrane, leading to higher energy consumption.^[^ ^15^ ^]^ Therefore, it is necessary to effectively regulate the structure and chemistry of NF membranes to obtain high‐performance membrane materials with better rejection of Mg^2+^ without sacrificing membrane permeability toward Li^+^ and water.

Surface post‐modification of the membrane provides a simple and efficient strategy to address the issues mentioned above. First, the post‐modification reaction will compete with the original PEI‐TMC amidation reaction, which may influence the structure and morphology of the separation layer, achieving better separation performance.^[^ ^16^ ^]^ Second, suitable modification monomers can be selected or designed to impart higher Li^+^ affinity to the membrane surface, facilitating Li^+^ permeability and further enhancing Li^+^/Mg^2+^ selectivity.^[^ ^12^, ^17^ ^]^ Oligoethers bearing crown ether‐like structures exhibit excellent cation affinity. The ion–dipole interactions between positively charged cations and negatively charged oxygen atoms allow lithium ions to be re‐solvated within the membrane after dehydration, thereby reducing the energy barrier for Li^+^ to cross the membrane.^[^ ^18^ ^]^ Therefore, introducing ether chain functional groups onto the membrane surface via post‐modification is a promising approach to achieving efficient lithium extraction from brine.

**Figure 1:**
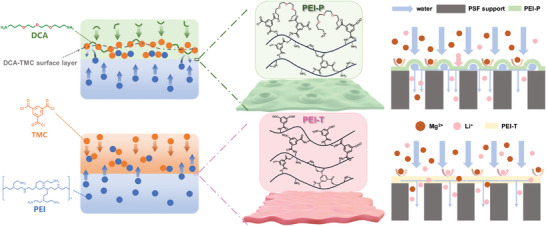
Schematic diagram of the reaction of PEI‐P and PEI‐T membranes. In particular, the PEI‐P membrane formed a unique coffee ring structure and a thinner active layer due to the DCA‐TMC layer at the top of the membrane that inhibited the upward diffusion of PEI. As a result, PEI‐P can better transport water and Li^+^ compared to PEI‐T membranes.

Herein, we employed the post‐modification approach to fabricate ether‐functionalized PEI nanofiltration membranes (PEI‐P). The nanofiltration membrane surface was successfully grafted with oligoether moieties through the amidation reaction between the amine groups of 4,7,10‐Trioxygen‐1,13‐tridecanediamine (DCA) and the acyl chloride functionalities in the PEI‐TMC. As a comparison, nanofiltration membranes with DCA as an in situ blended aqueous monomer (PEI‐I) were also prepared. Characterization and experimental results showed that the introduction of DCA not only resulted in the formation of a unique coffee ring structure on the membrane surface, but also the DCA‐TMC crosslinking structure limited the upward diffusion of PEI monomers and hindered the reaction between PEI and TMC monomers on the top of the membrane, which on the one hand, led to the reduction of the thickness of the membrane and enabled the membrane to maintain its excellent water permeability. On the other hand, the amine groups of the PEI monomer were retained, and the positive charge density of the membrane was increased, thus improving the rejection of Mg^2+^ (**Figure** **1**). Meanwhile, the affinity of the ether chain for Li^+^ ensures that the membrane can facilitate the transport of Li^+^. As a result, a high water flux (10.9 ± 0.2 L m^−2^ h^−1^ bar^−1^), a rapid Li^+^ permeation rate, and an excellent MgCl_2_ rejection (96 ± 1.6%) were achieved. Furthermore, the modified membranes exhibited a high Li^+^/Mg^2+^ selectivity (*S_Li,Mg_* = 23.3) in mixed salt tests, demonstrating their potential for extracting high‐purity Li_2_CO_3_ from feed solutions with high Mg^2+^/Li^+^ ratios.

## 2 Results and Discussion

### 2.1 Preparation and Characterization of NF Membranes

For unmodified PEI‐based nanofiltration membranes, PEI and TMC undergo interfacial polymerization on the surface of a polysulfone (PSF) ultrafiltration membrane to form an initial PEI‐TMC membrane (I‐PEI). The membrane is then subjected to further heating to promote a more complete polymerization of the monomers, resulting in the formation of the final PEI‐TMC membrane (PEI‐T). It is noteworthy that the I‐PEI membrane, owing to the absence of heat treatment, exhibits an abundance of unreacted acyl chloride moieties on its surface, enabling further modification of the membrane.^[^ ^19^ ^]^ Consequently, the amine groups present in DCA are employed to react with TMC, leading to the fabrication of PEI membranes functionalized with oligoether chains (PEI‐P). Simultaneously, PEI‐I membranes (PEI‐I), produced by the in situ blending of PEI and DCA followed by interfacial reaction with TMC, are also prepared for comparative analysis. The chemical composition of the PEI‐based nanofiltration membranes was characterized using Attenuated Total Reflection‐Fourier Transform Infrared Spectroscopy (ATR‐FTIR) and X‐ray Photoelectron Spectroscopy (XPS). As illustrated in Figure S2 (Supporting Information), the stretching vibration peak of the amide bond formed by the reaction between the amine group in PEI and the acyl chloride group in TMC appeared at 1645 cm^−1^, confirming the successful fabrication of the active polyamide layer.^[^ ^11^ ^]^ The characteristic peaks of ether bonds (1110 cm^−1^) were also shown in the FTIR spectra of the DCA monomers. However, the characteristic peaks of the ether bond in PEI‐P overlapped with the peaks of the other groups, so XPS characterization was used for further verification.^[^ ^20^ ^]^ The surface elemental composition of the membranes was further characterized by XPS. In comparison to PEI‐T, the N content on the surface of PEI‐P exhibits a slight decrease, while the C and O content, corresponding to the modifying monomer DCA, display a modest increase, substantiating the effective modification by the DCA monomer (Table S1, Supporting Information). Furthermore, the O content in PEI‐I also exhibits a notable increase. In the high‐resolution C1s XPS spectra, compared to the PEI‐T membrane, the PEI‐P membrane displays a characteristic peak of C‐O bonds at 286.3 eV,^[^ ^18a^ ^]^ while the content of C‐N bonds decreases from 36.3% to 14% (Figure S3a–c, Table S2, Supporting Information). Additionally, in the O1s spectra, the proportion of N‐C = O bonds (originating from the reaction between amine and acyl chloride groups) declines from 81% to 54.4%, which is consistent with the trends reported in the literature (Figure S3d–f, Table S3, Supporting Information).^[^ ^21^ ^]^ The decrease in the N element content and crosslink density on the membrane surface suggests that during the post‐modification process, the DCA monomers reacted with the TMC at the top of the membrane to form a crosslinked structure, which limited the upward diffusion of the PEI monomers and the reaction with the TMC, and thus may have led to the formation of a thinner selective layer.

To validate the aforementioned hypothesis, we tested the rejection of 1000 ppm MgCl_2_ solution by the unheated I‐PEI membrane at a pressure of 6 bar. The results show that the rejection of MgCl_2_ by I‐PEI is only 24% (Figure S4, Supporting Information). So we conclude that I‐PEI contains lots of unreacted PEI molecules, which need to be heated at 60 °C to form a dense PEI‐TMC layer. Electrostatic potential (ESP) was used to describe the reactivity of DCA and PEI molecules. PEI and DCA molecules are used as nucleophilic reagents, and the larger the negative electrostatic potential of the reaction site, the higher the reactivity. As shown in Figure S5 (Supporting Information), the maximum electrostatic potential in the PEI molecule is −46.09 kcal mol^−1^, which is lower than the ESP value of the amino group in the DCA molecule. Therefore, the DCA molecule has higher reactivity and will preferentially form a DCA‐TMC crosslinked structure at the top of the membrane. Finally, we conducted filtration experiments using an unheat‐treated DCA‐TMC crosslinked layer on PEI solutions with a concentration of 0.2 wt.%. The results showed that the total organic carbon value of the PEI solution decreased from 446 to 85.5 mg L^−1^ after filtration (Figure S6, Supporting Information), which indicated that the DCA‐TMC layer had a strong inhibition effect on the diffusion of PEI. Therefore, on the basis of the presence of numerous unreacted PEI molecules in I‐PEI, a spatially confined layer can be established by modifying the more active DCA molecules to preferentially form a DCA‐TMC cross‐linked structure at the top of the membrane, thereby restricting the PEI molecules to a narrower space and ultimately forming a thinner selective layer.

**Figure 2:**
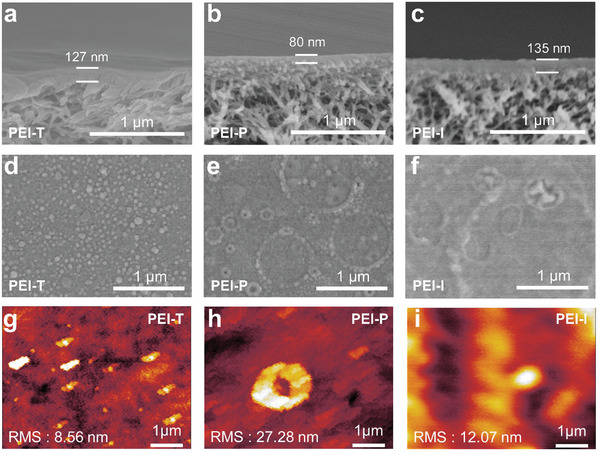
Cross‐sectional SEM images of a) PEI‐T, b) PEI‐P, and c) PEI‐I membranes. Top‐view SEM images of d) PEI‐T, e) PEI‐P, and f) PEI‐I membranes. AFM images of g) PEI‐T, h) PEI‐P, and i) PEI‐I membranes.

Cross‐sectional scanning electron microscopy (SEM) images were employed to characterize the membrane thickness. As illustrated in **Figure** **2**a–c, the thickness of the PEI‐P membrane is ≈80 nm, which is ≈35% thinner compared to the PEI‐T membrane. The cross‐sectional transmission electron microscopy (TEM) images also showed that the thickness of the PEI‐P membrane was 74.5 nm, which was much smaller than that of the PEI‐T membrane (117 nm), and the results were consistent with the SEM images (Figure S7, Supporting Information). In contrast, the thickness of the PEI‐I membrane increased to 112 nm. These observations further corroborate that the formation of DCA‐TMC cross‐linked structures on the membrane surface constrains the upward diffusion of PEI, resulting in a reduced membrane thickness. For all the NF membranes, the pores on the surface of the PSF substrate disappear due to the formation of the polyamide layer (Figure 2d–f; Figure S8, Supporting Information). The surface of the PEI‐T membrane exhibits a typical nodular structure, which is a characteristic morphology formed by the interfacial polymerization between PEI and TMC.^[^ ^14^, ^22^ ^]^ In the case of the PEI‐P membrane, coffee ring‐like structures emerge on the surface. The 2D and 3D AFM images also reveal changes in the roughness of the NF membranes. After the introduction of DCA monomers for post‐modification, the surface roughness of the PEI‐P membrane increased from 8.56 to 27.28 nm, and protruding ring‐like structures were detected (Figure 2g–i; Figure S9a,b, Supporting Information). This unique morphology is beneficial for increasing the filtration area.^[^ ^23^ ^]^ Surface profile images also showed the appearance of raised coffee ring‐like structures on the surface of the PEI‐P membrane (Figure S10, Supporting Information). In order to investigate the reason for the appearance of the coffee ring structure, the DCA‐TMC membrane was first prepared by reacting on the surface of the PSF substrate using an aqueous‐phase solution containing DCA and an oil‐phase solution containing TMC, which were thermally crosslinked. The top‐view SEM image shows that there is no rough structure formation on the surface of DCA‐TMC and the surface is relatively smooth (Figure S11a, Supporting Information). This suggests that the preparation of membranes using only DCA molecules cannot lead to the formation of the coffee ring structure. The DCA‐TMC membrane was then post‐modified using PEI molecules. The membrane surface still did not show any special structure (Figure S11b, Supporting Information). Therefore, we suggest that the more reactive DCA molecules reacted preferentially with TMC, while the post‐modified PEI molecules were not sufficient to affect the diffusion of DCA so that no structure was produced on the membrane surface. Furthermore, we post‐modified the I‐PEI membrane using a higher concentration of DCA solution (0.8 wt.%, denoted as PEI‐P‐8). Top‐view SEM images showed that the PEI‐P‐8 surface exhibited a regular pattern of Turing‐like structures (Figure S11c,d, Supporting Information).^[^ ^24^ ^]^ We speculated that it is because of the higher degree of cross‐linking of DCA with TMC, which creates a stronger resistance to the diffusion of PEI, thus leading to the creation of the special structure. The above results illustrate the important role of post‐modification of membranes using DCA monomers for the appearance of the coffee ring structure. To the best of our knowledge, diffusion of the solvent may have led to the enrichment of the reactive monomers at the edges of the solvent, resulting in a coffee ring structure.^[^ ^25^ ^]^ Accordingly, we give possible reasons for the formation of the coffee ring structure. During the process of interfacial polymerization, the upward diffusion of PEI molecules is inhibited due to the preferential formation of the top DCA‐TMC crosslinked structure. During heat treatment, the PEI molecules move faster, diffuse, and enrich toward the edges of the DCA‐TMC crosslinked structure, and further react with the TMC molecules to form the coffee ring structure (Figure S11e, Supporting Information).

**Figure 3:**
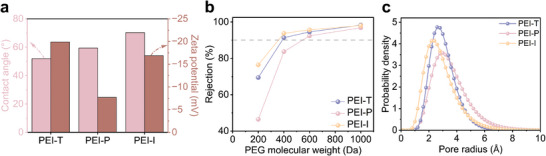
a) Static water contact angles and zeta potential values under neutral conditions for PEI‐T, PEI‐P, and PEI‐I membranes. b) Rejection of PEG molecules by PEI‐T, PEI‐P, and PEI‐I membranes. c) Pore size distributions of PEI‐T, PEI‐P, and PEI‐I membranes.

After incorporating the DCA monomer, the hydrophilicity of the PEI‐P membrane slightly decreased due to the increased alkyl content on the membrane surface (**Figure** **3**a; Figure S12, Supporting Information). However, the PEI‐P membrane exhibited higher positive charge density owing to the increased unreacted amine functional groups. Compared to the PEI‐T membrane, the zeta potential of the PEI‐P membrane increased from −19.9 to −7.7 mV, enabling enhanced rejection performance for MgCl_2_. The average pore size distribution of the membranes was calculated based on the rejection of PEG molecules. As illustrated in Figure 3b, the molecular weight cut‐off (MWCO) of the PEI‐T membrane was significantly lower than that of the PEI‐P membrane. This can be attributed to the competition between DCA and PEI, resulting in a reduced degree of crosslinking. Consequently, the PEI‐P membrane possessed a larger average pore size (Figure 3c).^[^ ^26^ ^]^

### 2.2 Separation Performance of NF Membrane

The water permeability and rejection performance of the NF membranes for MgCl_2_ and LiCl were evaluated using a cross‐flow filtration setup at an operating pressure of 6 bar. The membrane fabrication methods and conditions were optimized based on these results. Figure S13a (Supporting Information) illustrates the influence of different membrane preparation techniques on the separation performance of the membranes. We first examined the rejection of the membranes for LiCl (*R_LiCl_*). It was observed that the PEI‐T membrane exhibited a relatively high rejection for LiCl. However, upon introducing oligoether chains with high affinity for Li^+^, the rejection of LiCl by the PEI‐P membrane decreased from 63.8 ± 2.9% to 54.2 ± 2.5%. The PEI‐I membrane, which possessed a higher ether content, demonstrated an even lower rejection for LiCl. Therefore, the introduction of oligoether chains is important for accelerating the transmembrane transport of Li^+^. In the case of MgCl_2_, the PEI‐P membrane demonstrated an increase in rejection (*R_MgCl2_*) from 89.7 ± 0.9% to 96.2 ± 1.6% compared to the PEI‐T membrane, while maintaining membrane permeability (Figure S13b, Supporting Information). Although the in situ blended PEI‐I membrane also exhibited enhanced rejection capability for MgCl_2_, the water flux decreased by a factor of 2.5 due to the increased crosslinking density of the separation layer. These findings suggest that the PEI‐based nanofiltration membranes modified with oligoether chains via post‐modification can achieve rapid cation separation while maximally preserving membrane permeability.

**Figure 4:**
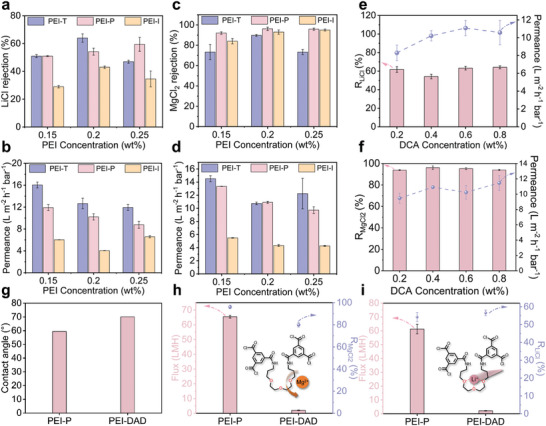
The rejection and water permeability of PEI‐T, PEI‐P, and PEI‐I membranes prepared with different concentrations of PEI solutions were tested with a,b) 1 g L^−1^ LiCl and c,d) 1 g L^−1^ MgCl_2_ aqueous solutions. The rejection and water permeability of PEI‐P membranes prepared with different concentrations of DCA solutions were tested with e) 1 g L^−1^ LiCl and f) 1 g L^−1^ MgCl_2_ aqueous solutions. g) Comparison of static water contact angles of PEI‐P and PEI‐DAD membranes. The separation performance of PEI‐P and PEI‐DAD membranes were compared using 1 g L^−1^ h) MgCl_2_ and i) LiCl aqueous solutions at a pressure of 6 bar. Error bars in (a–f,h,i) represent the SD (*n* = 3) and data are presented as mean values ± SD.

We further investigated the influence of PEI concentration on membrane performance. As the PEI concentration increased, the crosslinking density of the membranes also increased. Consequently, both PEI‐P and PEI‐I membranes exhibited improved rejection performance for MgCl_2_ and LiCl (**Figure** **4**a,c). However, due to the limited concentration of TMC, excessively high PEI concentrations resulted in a separation layer with lower crosslinking density, resulting in a decrease in *R_MgCl2_*.^[^ ^17^ ^]^ In comparison to the other two types of membranes, the PEI‐I membrane experienced a drastic decline in water flux (Figure 4b,d). Considering the Li^+^/Mg^2+^separation efficiency and permeability, we selected a 0.2 wt.% PEI aqueous solution and employed the post‐modification method to fabricate oligoether‐functionalized NF membranes. Furthermore, PEI‐P membranes modified with different DCA concentrations were investigated. As illustrated in Figure 4e,f, with increasing DCA modification concentration, the rejection of PEI‐P for MgCl_2_ initially increased and then reached saturation. In contrast, the rejection for LiCl exhibited a trend of initial decline followed by an increase. This is because of the simultaneous promotion of Li^+^ by oligoether chains and the hindrance of Li^+^ by size sieving in PEI‐P membranes. With the increase of DCA modification, the content of ether chains in the PEI‐P membrane increased, but the crosslink density also increased, which led to the increase of size sieving effect (Figure S14, Table S4, Supporting Information). The hindering effect of size sieving on Li^+^ permeation gradually exceeded the promoting effect of oligo ether chains on Li^+^, so that the rejection of Li^+^ by PEI‐P showed a decreasing and then increasing trend. Overall, the water flux of the membranes was not significantly affected by the DCA concentration. The slight increase in water flux is on the one hand due to the increase in membrane hydrophilicity (Figure S15, Supporting Information), and on the other hand due to the appearance of Turing‐like structural patterns on the membrane surface, which increase the permeation area. Therefore, the optimal modification concentration of DCA was determined to be 0.4 wt.%. Under this condition, the affinity of the oligoether chains for lithium ions facilitated the transport of Li^+^, while the electrostatic repulsion and steric hindrance effects maximally impeded the passage of Mg^2+^.

To further validate that the affinity of oligoether chains for Li^+^ can facilitate lithium‐ion transport, we designed 1,12‐Diaminododecane (DAD) as a control monomer, which possesses a structure analogous to the DCA monomer but devoid of ether chains, to post‐synthetically modify the I‐PEI membrane. As depicted in Figure S16 (Supporting Information), the characteristic peaks of amide bonds appeared in the FTIR spectra of PEI‐DAD, which proved the successful preparation of the membrane. The introduction of DAD led to an increase in the peak area of C = C/C‐C bonds on the surface of the PEI membrane, as evidenced by the C1s spectrum. Conversely, compared to PEI‐T, the O1s spectra showed a reduction in the peak area of the N‐C = O bonds of the PEI‐DAD membrane, indicating a decrease in the degree of cross‐linking of the membrane. The pore size distribution of PEI‐DAD was also measured, and the results showed that the pore size distribution of PEI‐DAD was wider than that of PEI‐P membrane (Figure S17, Supporting Information). The alkane chains contained in the DAD monomer resulted in a decrease in the hydrophilicity of the PEI‐DAD membrane (Figure 4g). The salt rejection performance of the PEI‐DAD membrane was evaluated using 1000 ppm MgCl_2_ and LiCl solutions. Figure 4h,i illustrates that the PEI‐DAD membrane exhibited a rejection of merely 80 ± 2.1% for Mg^2+^, significantly lower than that of the PEI‐P membrane, accompanied by a drastic decrease in flux from 65.5 ± 0.95 L m^−2^ h^−1^ (LMH) to 2.1 ± 0.21 LMH. Interestingly, the rejection of the PEI‐DAD membrane for Li^+^ surpassed that of the PEI‐P membrane. These findings demonstrate that the ion–dipole interactions between oligoether chains and Li^+^ enable rapid permeation of Li^+^ through the NF membrane while effectively rejecting Mg^2+^. Furthermore, the rough surface structure and thinner separation layer induced by the post‐modification of the DCA monomer contribute to the enhancement of membrane permeability.

**Figure 5:**
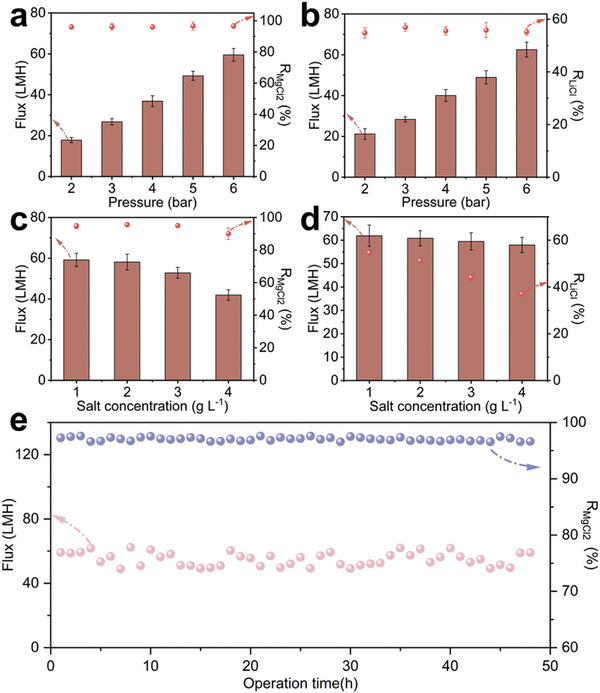
The separation performance of PEI‐P membranes for a) MgCl_2_ b) LiCl aqueous solutions of 1 g L^−1^ at different pressures. Effect of c) MgCl_2_ and d) LiCl concentration in the separation performance of PEI‐P membrane at 6 bar pressure. e) Nanofiltration stability of PEI‐P membrane. Error bars in (a–d) represent the SD (*n *= 3) and data are presented as mean values ± SD.

The stability of the membrane is of particular importance in practical operation. We first tested the separation performance of PEI‐P under different pressures. As shown in **Figure** **5**a,b, the water flux of PEI‐P exhibited a linear relationship with the operating pressure for an aqueous MgCl_2_ solution (1000 ppm), and the rejection is maintained above 95%. The rejection for LiCl is maintained at 54%, demonstrating good pressure stability. The tolerance of the membrane to high salt conditions also affects its practical application performance. As the concentrations of MgCl_2_ and LiCl increase from 1000 to 4000 ppm, the osmotic pressure difference between the feed solution and the permeate increases, leading to a decrease in the flux of the membrane (Figure 5c,d). However, the rejection for MgCl_2_ can be maintained above 90%, while *R_LiCl_* decreases from 54 ± 1% to 37 ± 0.6%, indicating the potential of PEI‐P for extracting lithium resources from solutions with high Mg/Li ratios. Furthermore, PEI‐P is capable of maintaining high separation performance under continuous nanofiltration conditions for 48 h (Figure 5e).

### 2.3 Lithium Separation Process and Performance

**Figure 6:**
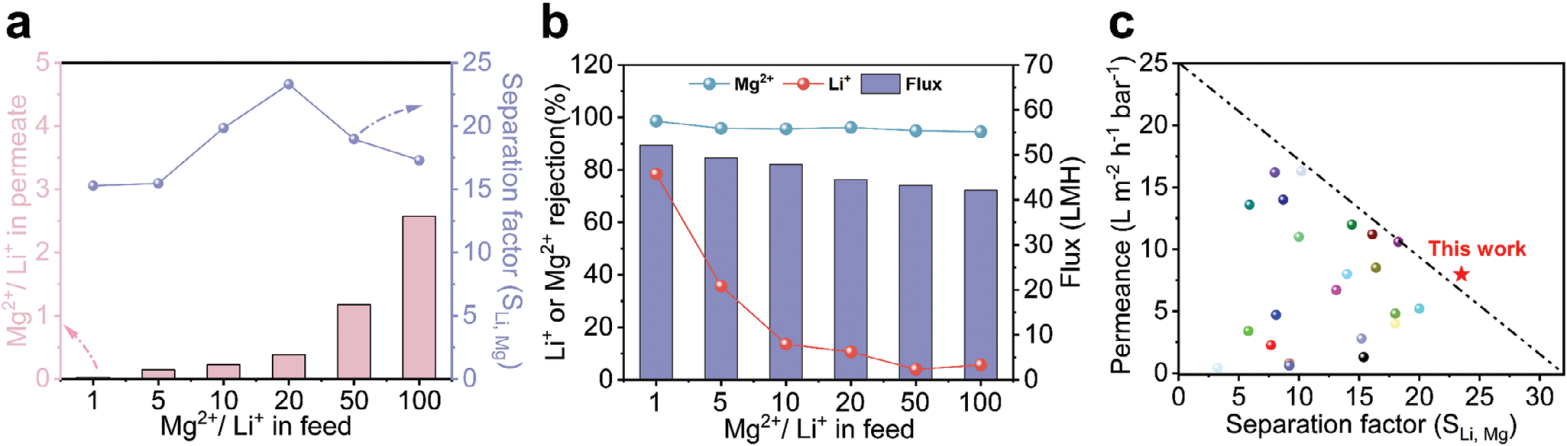
a) Separation factor b) salt rejection, and water flux of PEI‐P membranes for feed solutions with different Mg^2+^/Li^+^ ratios (all feeds total salt concentration was kept at 2000 ppm, pressure: 6 bar). c) Comparison of Li^+^/Mg^2+^ separation performance of PEI‐P membrane with other membranes in the literature.

Various simulated salt solutions with different Mg^2+^/Li^+^ mass ratios were employed to verify the potential of PEI‐P for extracting lithium under high Mg^2+^/Li^+^ ratio conditions. As illustrated in **Figure** **6**a,b, with the increase in the Mg^2+^/Li^+^ ratio of the feed solution, the separation factor (*S_Li,Mg_*) of PEI‐P can be maintained above 15. When the Mg^2+^/Li^+^ ratio is 20, the Li^+^/Mg^2+^ selectivity of the PEI‐P membrane reaches 23.3. Even at high Mg^2+^/Li^+^ ratios, the PEI‐P membrane is capable of maintaining ≈95% rejection for MgCl_2_. In contrast, Li^+^ can be rapidly adsorbed and transferred within the membrane due to its higher affinity with the oligoether chains. Compared to previously reported Li^+^/Mg^2+^ separation membranes, PEI‐P exhibits excellent performance in terms of both permeability and Li^+^/Mg^2+^ selectivity (Figure 6c, Table S5, Supporting Information).^[^ ^10^, ^11^, ^12^, ^14^, ^17^, ^19^, ^22^, ^27^ ^]^

**Figure 7:**
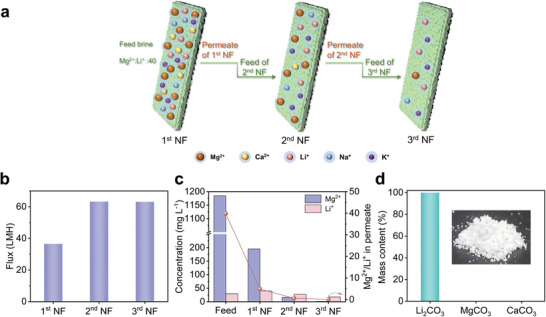
a) Schematic illustration of a three‐stage nanofiltration process for lithium extraction from a simulated salt lake brine solution consisting of 183 mg L^−1^ LiCl, 4700 mg L^−1^ MgCl_2_, 609 mg L^−1^ NaCl, 457 mg L^−1^ KCl, and 124 mg L^−1^ CaCl_2_. b) Water flux of PEI‐P membrane in three‐stage nanofiltration process. c) Mg^2+^/Li^+^ mass ratio and Li^+^ and Mg^2+^ concentrations in the feed solution and permeate for each stage of the NF process. d) Mass content of Li_2_CO_3_, MgCO_3_, and CaCO_3_ in the product after purification, the inset image is a digital photo of the Li_2_CO_3_ product.

Under binary salt conditions, PEI‐P exhibited excellent lithium extraction performance. However, it has been reported that neglecting the transport of Na^+^ and K^+^ can lead to an overestimation of the Li^+^/Mg^2+^ selectivity of the membrane by 250%.^[^ ^28^ ^]^ Therefore, to verify the practical application potential of PEI‐P for lithium extraction from salt lakes, we simulated the ionic composition of Dongtai Genel salt lake and prepared simulated lake water containing multiple ions (K^+^, Na^+^, Ca^2+^, Li^+^, Mg^2+^, Cl^−^), with a Mg^2+^/Li^+^ mass ratio of 40. A three‐stage nanofiltration process was employed for lithium extraction. As illustrated in **Figure** **7**a, the permeate obtained from the previous stage of the nanofiltration separation process was utilized as the feed solution for the subsequent stage. During the initial nanofiltration stage, the PEI‐P membrane exhibited lower permeability (36.6 LMH) due to the higher osmotic pressure. As the salt concentration of the simulated lake water progressively decreased through nanofiltration, the permeability of the PEI‐P membrane ultimately increased to 63 LMH (Figure 7b). After three nanofiltration stages, the concentration of Mg^2+^ in the simulated salt lake was reduced from 1200 to 1.6 mg L^−1^, while the concentration of Ca^2+^ was below the detection limit and could not be detected. In contrast, the concentration of Li^+^ only decreased from an initial value of 29.8 to 17.8 mg L^−1^. The final permeate achieved a Mg^2+^/Li^+^ ratio of 0.09, paving the way for the subsequent acquisition of high‐purity lithium resources (Figure 7c). Furthermore, to prepare a high‐purity Li_2_CO_3_ product, we initially added NaOH to the permeate obtained from the third nanofiltration stage and centrifuged the solution to remove trace amounts of Mg^2+^. Taking advantage of the fact that the solubility of Li_2_CO_3_ decreases with increasing temperature, we introduced high‐purity CO_2_ into the solution at 80 °C, resulting in the precipitation of Li_2_CO_3_. The precipitate was washed with high‐temperature deionized water and dried. ICP analysis revealed that the purity of the obtained Li_2_CO_3_ exceeded 99.5% (Figure 7d). We also tested the FTIR spectra of PEI‐P membranes before and after nanofiltration. The results showed that the chemical composition of the PEI‐P membrane had no significant change before and after the test, proving the actual stability of the membrane (Figure S18, Supporting Information). These results demonstrate the potential of PEI‐P membranes for extracting lithium resources from various types of salt lake water.

## 3 Conclusion

A PEI‐P membrane with excellent Li^+^/Mg^2+^ separation performance was prepared by surface modification of PEI‐TMC membrane using DCA monomer containing oligoether‐based chains by the post‐modification method. Characterization and experimental results showed that the crosslinked structure formed by DCA and TMC at the top of the membrane hindered the upward diffusion of PEI, thus achieving a thinner selective layer and a higher positive charge density. The highly lithophilic oligoether chain structure promotes the adsorption of lithium ions on the membrane surface and the transportation process within the membrane. Consequently, the PEI‐P membrane exhibited an exceptional rejection for Mg^2+^ (*R_MgCl2_* = 96 ± 1.6%) and a high water permeability (10.9 ± 0.2 L m^−2^ h^−1^ bar^−1^). During the mixed salt separation process, PEI‐P also demonstrated excellent lithium–magnesium separation performance (*S_Li,Mg_* = 23.3). In addition, PEI‐P membranes were used to extract lithium from a simulated salt lake with a Mg^2+^/Li^+^ ratio of 40, and Li_2_CO_3_ products with a purity of exceeding 99.5% were successfully prepared, which showed a promising application prospect in the actual salt lake lithium extraction industry.

## 4 Experimental Section

### Materials

Trimesoyl chloride (TMC, 98%), poly (ethyleneimine) (PEI, M_w_: 70 000, 50 wt.% in water), 4,7,10‐Trioxygen‐1,13‐tridecanediamine (DCA, 97%), Polyethylene glycol (PEG), KCl, NaCl, LiCl, MgCl_2_, CaCl_2_, sodium dodecyl sulfate (SDS) were bought from the Aladdin Biochemical Technology Co., Ltd. (Shanghai, China). *n*‐Hexane (AR) was provided by Meryer Chemical Technology Co., Ltd. (Shanghai, China). The conductivity of deionized (DI) water used in all experiments was less than 2 µS cm^−1^. Polysulfone (PSF) substrate with an MWCO (20 000 Da) was obtained from Guochu Technology Co., Ltd. (Xiamen, China).

### Membrane Preparation—PEI‐T Membrane

The PEI‐T membrane was fabricated via interfacial polymerization between PEI and TMC. A PEI aqueous solution (0.2 wt.%) was prepared by dissolving PEI (0.1 g) and SDS (0.05 g) in deionized water (50 g). Concurrently, a TMC organic phase (0.1 wt.%) was obtained by dissolving TMC (0.05 g) in *n*‐hexane (50 g). The interfacial polymerization method was employed for the preparation of the PEI‐T membrane. Initially, the PEI aqueous solution was poured onto the surface of a PSF substrate membrane and allowed to soak for 5 min. Subsequently, the excess solution was drained off, and the membrane was dried at room temperature until no visible water droplets remained on the surface. Subsequently, the membrane surface was covered with the TMC solution for 30 s, after which the excess TMC solution was carefully removed, resulting in the formation of the membrane denoted as I‐PEI. After air‐drying for 3 min, the membrane was heated in an oven at 60 °C for 10 min to obtain the PEI‐T membrane. Prior to use, the membranes were stored in deionized water. Note: Unless otherwise specified, all PEI‐based NF membranes were prepared using a 0.2 wt.% PEI aqueous solution.

### Membrane Preparation—PEI‐I Membrane

The PEI‐I membrane was prepared using an in situ blending approach. The fabrication process was similar to that of the PEI‐T membrane, with the exception that the PEI aqueous solution was replaced by a PEI‐DCA mixed solution, which was obtained by dissolving PEI (0.2 g), SDS (0.05 g), and DCA (0.2 g) in of deionized water (50 mL).

### Membrane Preparation—PEI‐P Membrane

As illustrated in Figure S1 (Supporting Information), the PEI‐P membrane was fabricated via a post‐modification method. After allowing the I‐PEI membrane to dry at room temperature for 1 min, the surface was wetted with an aqueous DCA solution (0.4 wt.%) for 30 s, followed by the removal of excess water from the membrane surface. The membrane was then dried in air for 3 min before being placed in an oven for heating (60 °C, 10 min) to form the PEI‐P membrane. Prior to use, the membrane was stored in deionized water. Note: Unless otherwise specified, the concentration of the DCA monomer used for post‐modification was consistently maintained at 0.4 wt.%.

### Membrane characterization

Scanning electron microscopy (SEM) was performed on a Hitachi SU 8010. ATR‐FTIR (Nicolet iS10) was used to record FTIR spectra of the membrane. The inner chemistry of membranes was characterized through XPS analysis (Thermo Scientific K‐Alpha). Contact angles of water were measured on a contact angle measuring system SL200KB (USA KNO Industry Co.), equipped with a CCD camera. The static contact angles were measured in sessile drop mode. Inductively coupled plasma‐mass Spectrometry (ICP‐MS, PerkinElmer NexION 300X) was used to test the ionic concentration of the feed and permeate mixed salt solution.

### Determination of the Pore Size and Pore Size Distribution of Membranes

Neutral organic molecules, polyethylene glycols (PEGs), were employed to evaluate the pore size of the membranes. Aqueous solutions containing 100 ppm of PEG‐200, PEG‐400, PEG‐600, and PEG‐1000 were used to determine the membrane pore size under a filtration pressure of 4 bar. The concentration of PEG in the permeate was assessed using the UV‐barium chloride method.^[^ ^29^ ^]^ Prior to testing, the samples were diluted to a specific concentration to ensure that the absorbance fell within the range of 0.2–0.8. Subsequently, 1 mL of 0.05 M iodine standard solution and 1.2 mL of 5% barium chloride solution were added to 5 mL of the sample. After allowing the color to develop for 10 min, the absorbance was measured at a wavelength of 610 nm, and the concentration of PEG was calculated accordingly.

The Stokes diameter (*d_s_*, nm) of PEG molecules could be calculated from the following equations:

(1) $$d_{s}=\;33.5\times 10^{-3}\times M_{w}^{0.557}$$

The relationship between PEG rejection (R) and molecular weight was fitted using a linear log‐normal probability function. The pore size distribution was then calculated from the following probability density function:

(2) $$\frac{dR\left(r_{p}\right)}{d\left(r_{p}\right)}\;=\;\frac{1}{r_{p}\ln\sigma_{p}\;\sqrt{2\pi }}\;exp\left[-\frac{{\left(lnr_{p}\;-\;ln\mu_{p}\right)}^{2}}{2{\left(ln\sigma_{p}\right)}^{2}}\right]$$

where μ_*p*_ is the mean effective pore size of the membrane, which equals the Stokes radius when *R*  = 50%; σ_*p*_ is defined as the geometric standard deviation of the membrane pore size, which is the ratio of the Stokes diameter corresponding to *R* = 83.14% of the PEG molecule to μ_*p*_.

### Membrane Performance Evaluation

The performance of the nanofiltration membranes was evaluated using a cross‐flow filtration setup with an effective membrane area of ≈7.1 cm^2^. The feed solution contained salt ions at a concentration of 1 g L^−1^, and the operating pressure was maintained at 6 bar. To ensure the accuracy of the experiments, all membranes were subjected to a 30‐min stabilization period at 6 bar before collecting the permeate. The permeate flux of the membranes was calculated by measuring the mass of the permeate collected over time. The ion rejection and ion selectivity were determined by measuring the conductivity of the feed solution and the permeate. Each sample was tested in triplicate to ensure reproducibility.

Salt rejection was calculated using Equation (3), where *C_f_* and *C_p_* denote the conductivity of the feed solution and permeate, respectively.

(3) $$R\;\;=\;\left(1\;-\;\frac{C_{P}}{C_{f}}\right)\;\;\times \;100\%$$

The flux *J* was determined using the Equation (4):

(4) $$\begin{array}{c} J\;\;=\;\frac{W}{\rho At}\; \end{array}$$

where *A* is the effective membrane area, ρ is the density of the permeate, and *W* is the mass of the permeate collected over a given time *t*. For mixed salt solutions, the concentrations of individual ions were determined using inductively coupled plasma mass spectrometry (ICP‐MS). Li^+^/Mg^2+^ separation factor (*S_Li,Mg_*) was calculated by the following equation:

(5) $$S_{Li,Mg}\;=\;\frac{C_{p,Li^{+}}/C_{p,Mg^{2+}}}{C_{f,Li^{+}}/C_{f,Mg^{2+}}}$$

where $C_{f,Li^{+}}$, $C_{f,Mg^{2+}}$, $C_{p,Li^{+}}$, $C_{p,Mg^{2+}}$ are the concentrations of LiCl and MgCl_2_ in feed and permeate solution.

### Statistical Analysis

Nanofiltration tests were replicated independently at least three times for all single‐salt solutions. Data were expressed as mean ± standard deviation (SD) and sample size (n) for each statistical analysis was represented in the corresponding figure legends.

## Conflict of Interest

The authors declare no conflict of interest.

## Supporting information

Supporting Information

## Data Availability

The data that support the findings of this study are available from the corresponding author upon reasonable request.
