# Supplementary material for: Space‐Confined Synthesis of Thinner Ether‐Functionalized Nanofiltration Membranes with Coffee Ring Structure for Li+/Mg2+ Separation
Source: Adv Sci (Weinh). 2024 Sep 13;11(41):2404150. doi: 10.1002/advs.202404150 (PMC11538659; doi:10.1002/advs.202404150)
Supplement: Supplementary file 1 — Supporting Information [file ADVS-11-2404150-s001.docx]

Supporting Information

**Space-Confined Synthesis of Thinner Ether-Functionalized Nanofiltration Membranes with Coffee Ring Structure for Li^+^/Mg^2+^ Separation**

Wentong Meng,^a^ Sifan Chen,^a^ Pu Chen,^a^ Feng Gao,^a^ Jianguo Lu,^b^ Yang Hou,^a^ Qinggang He,^a^ Xiaoli Zhan,^a^ Qinghua Zhang ^a,^*


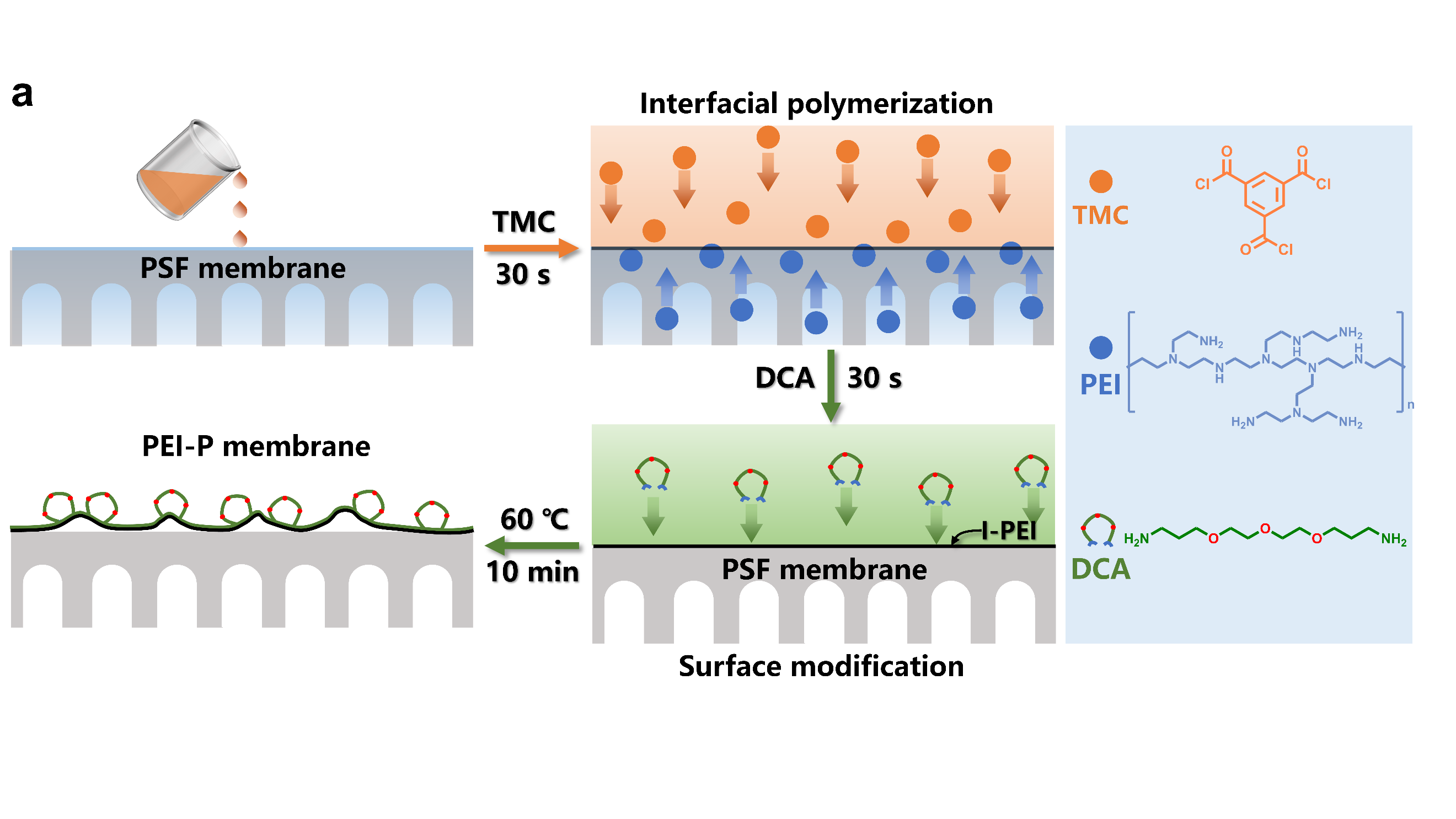


**Figure S1.** (a) Schematic diagram of the preparation of PEI-P membrane.


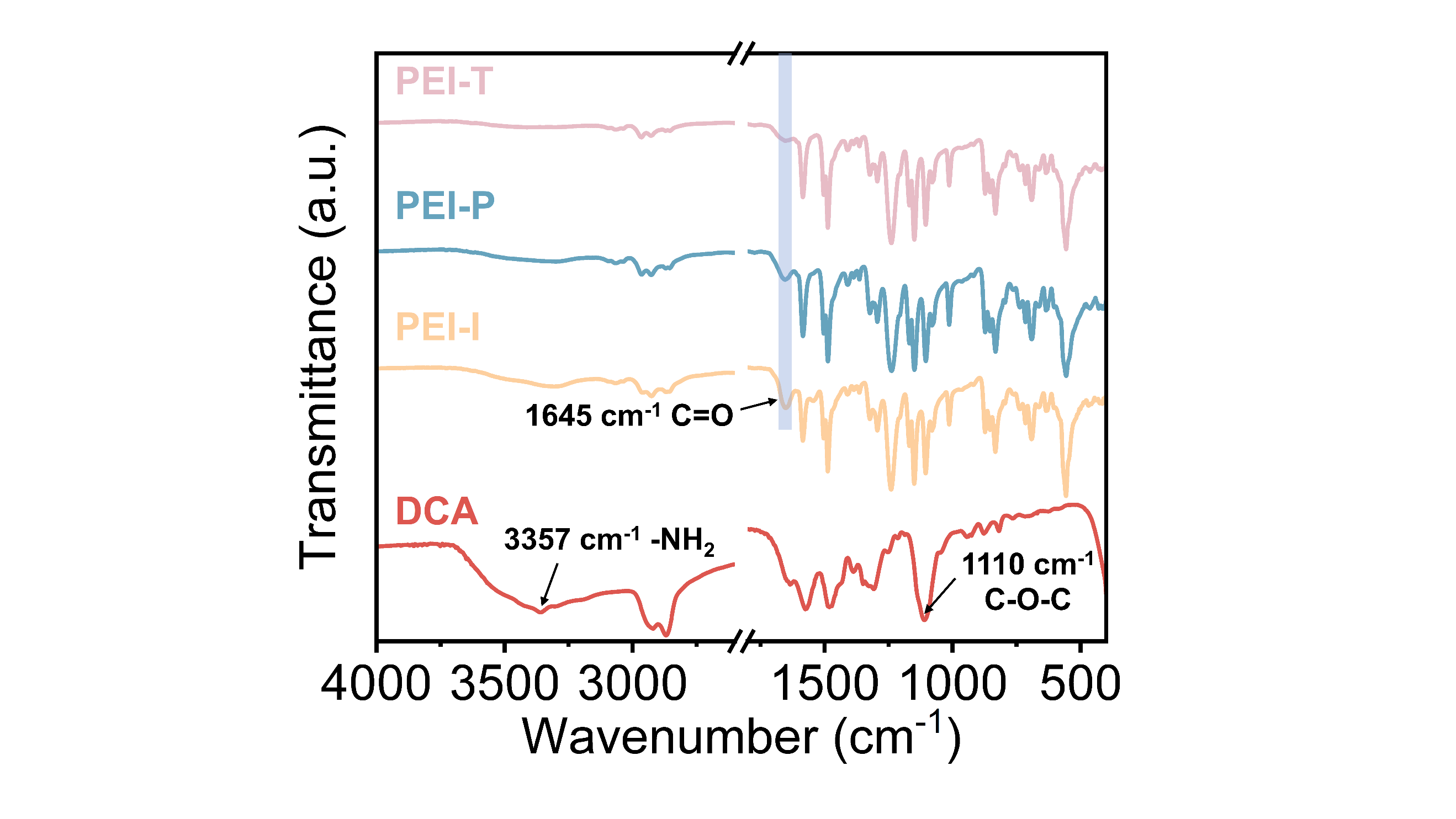


**Figure S2.** FTIR spectra of PEI-T, PEI-P, PEI-I membranes and DCA monomers.


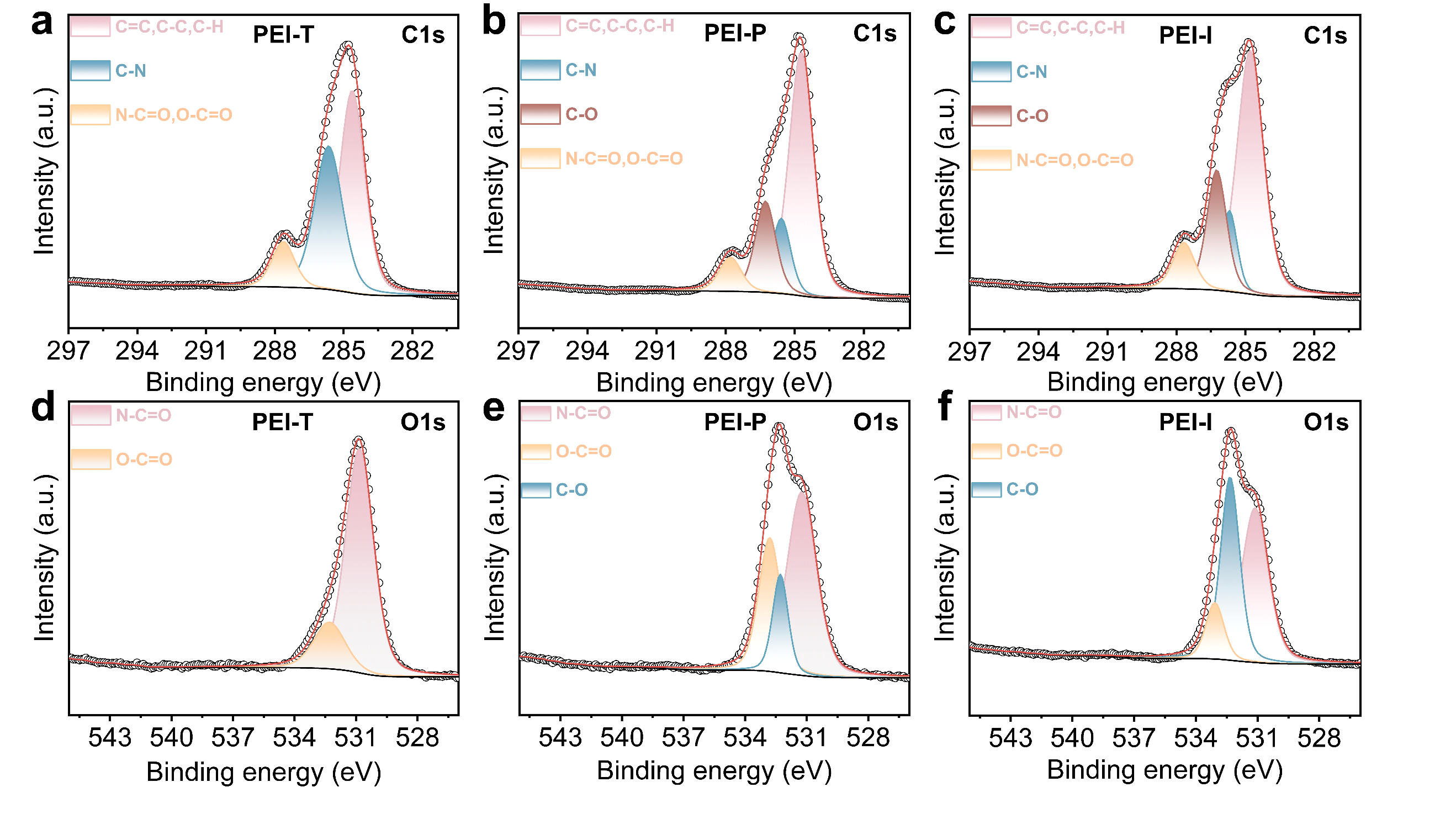


**Figure S3.** (a-c) High-resolution C1s XPS spectra of PEI-T, PEI-P, and PEI-I membranes. (d-f) High-resolution O1s XPS spectra of PEI-T, PEI-P, and PEI-I membranes.


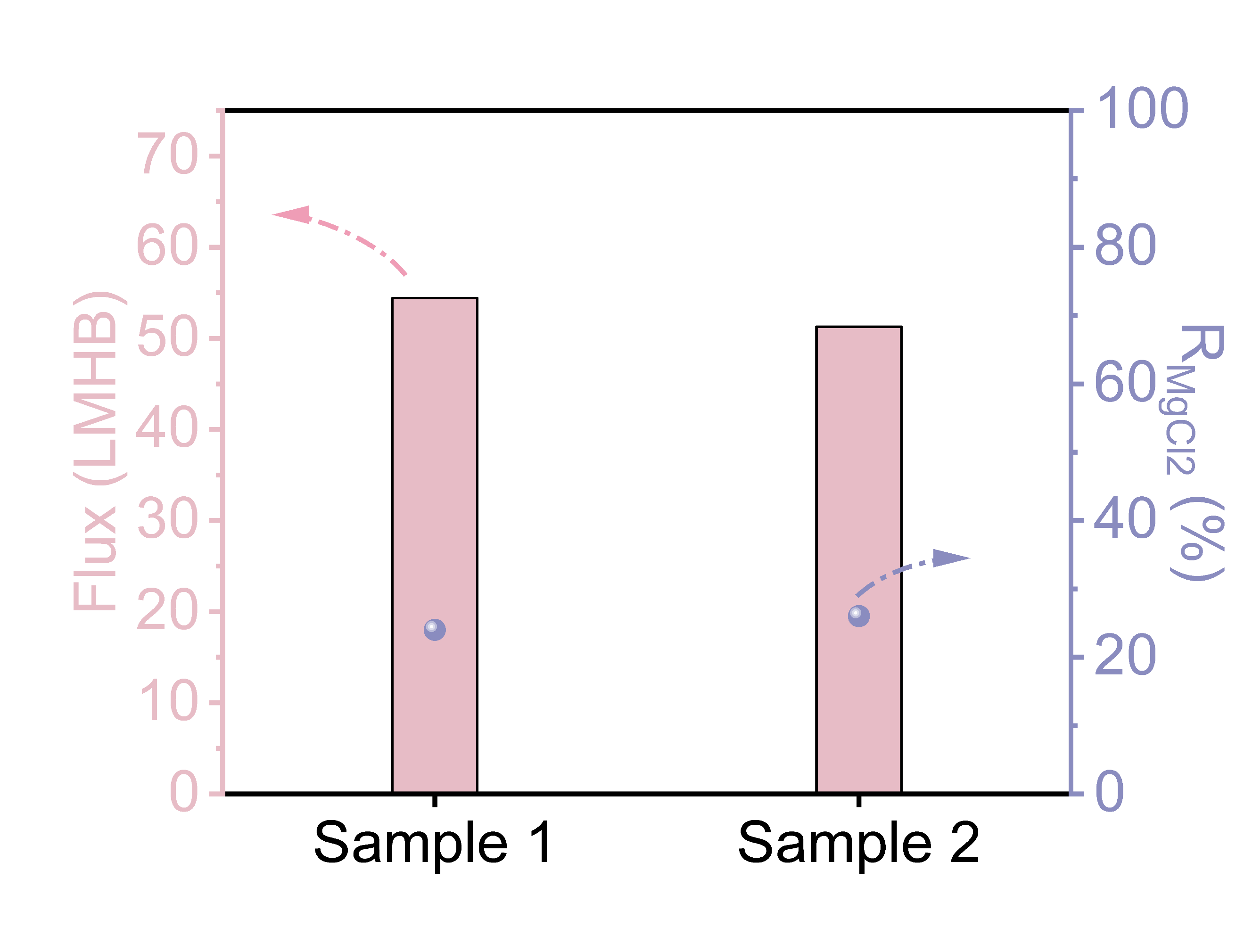


**Figure S4.** The rejection of I-EPI membrane to 1000 ppm MgCl_2_ solution was tested at 6 bar pressure. Where sample 1 and sample 2 are I-PEI membranes prepared under the same conditions.


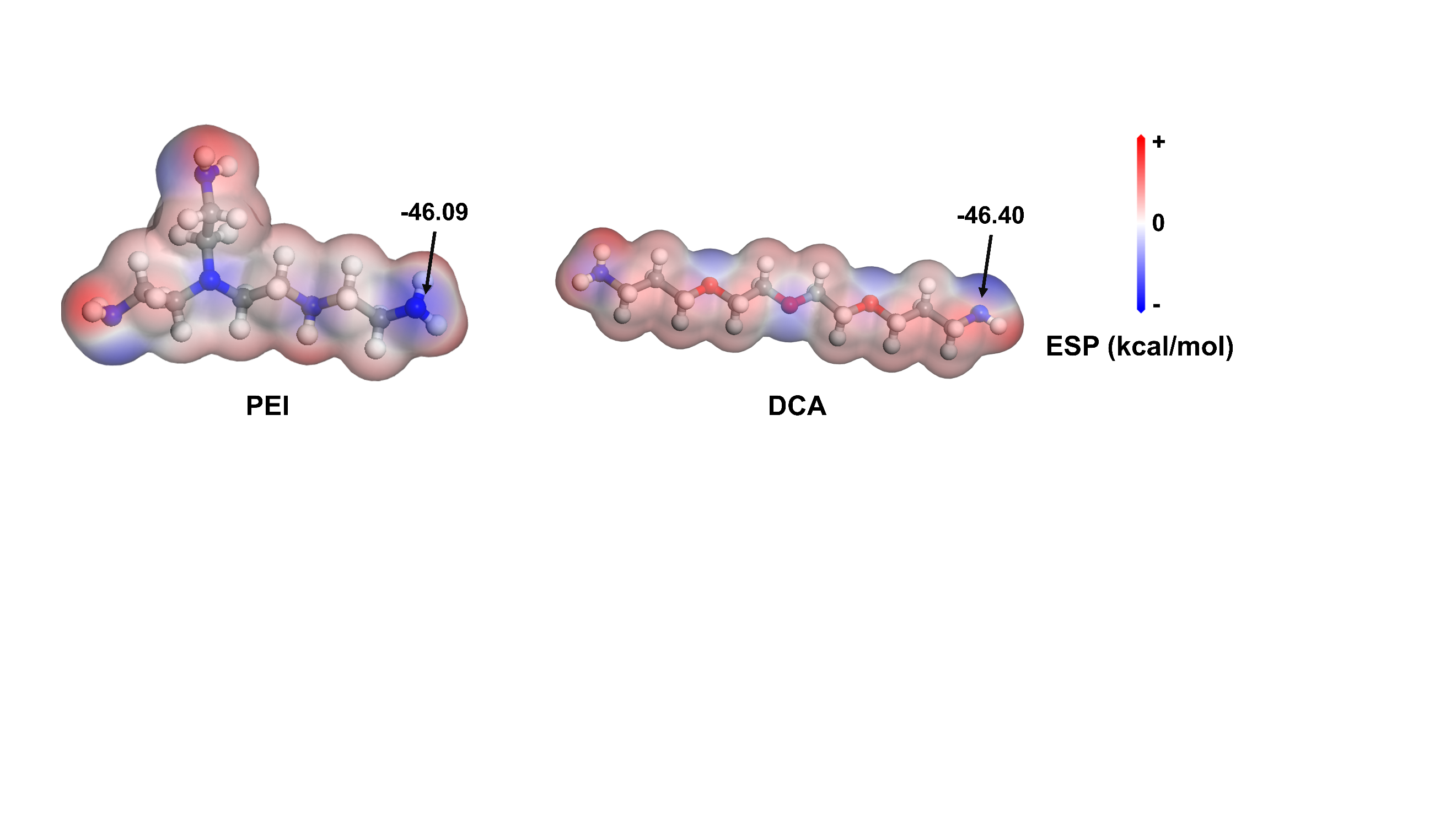


**Figure S5.** ESP-mapped molecular van der waals surface of PEI, DCA.


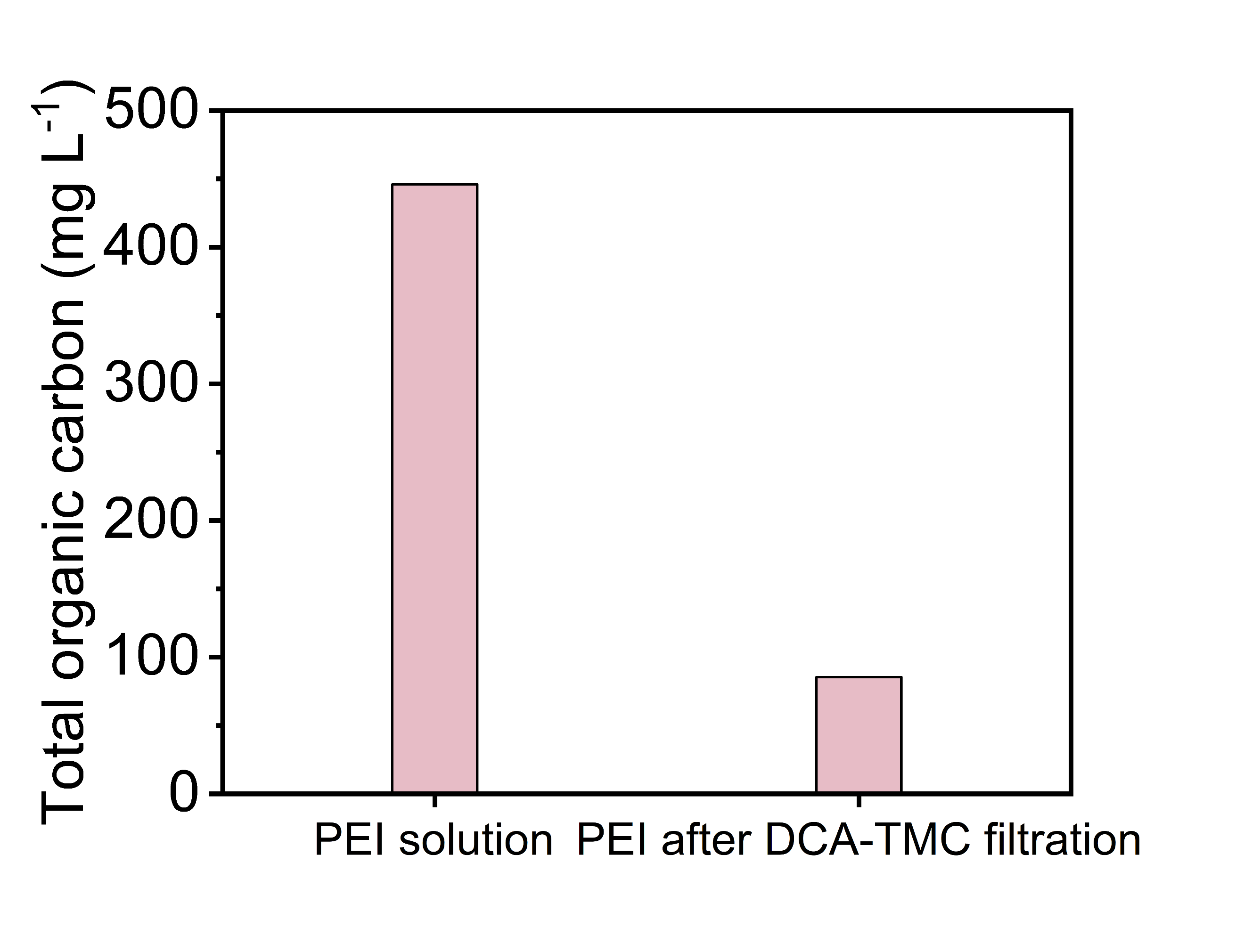


**Figure S6.** TOC values of PEI solutions before and after nanofiltration by DCA-TMC layer.


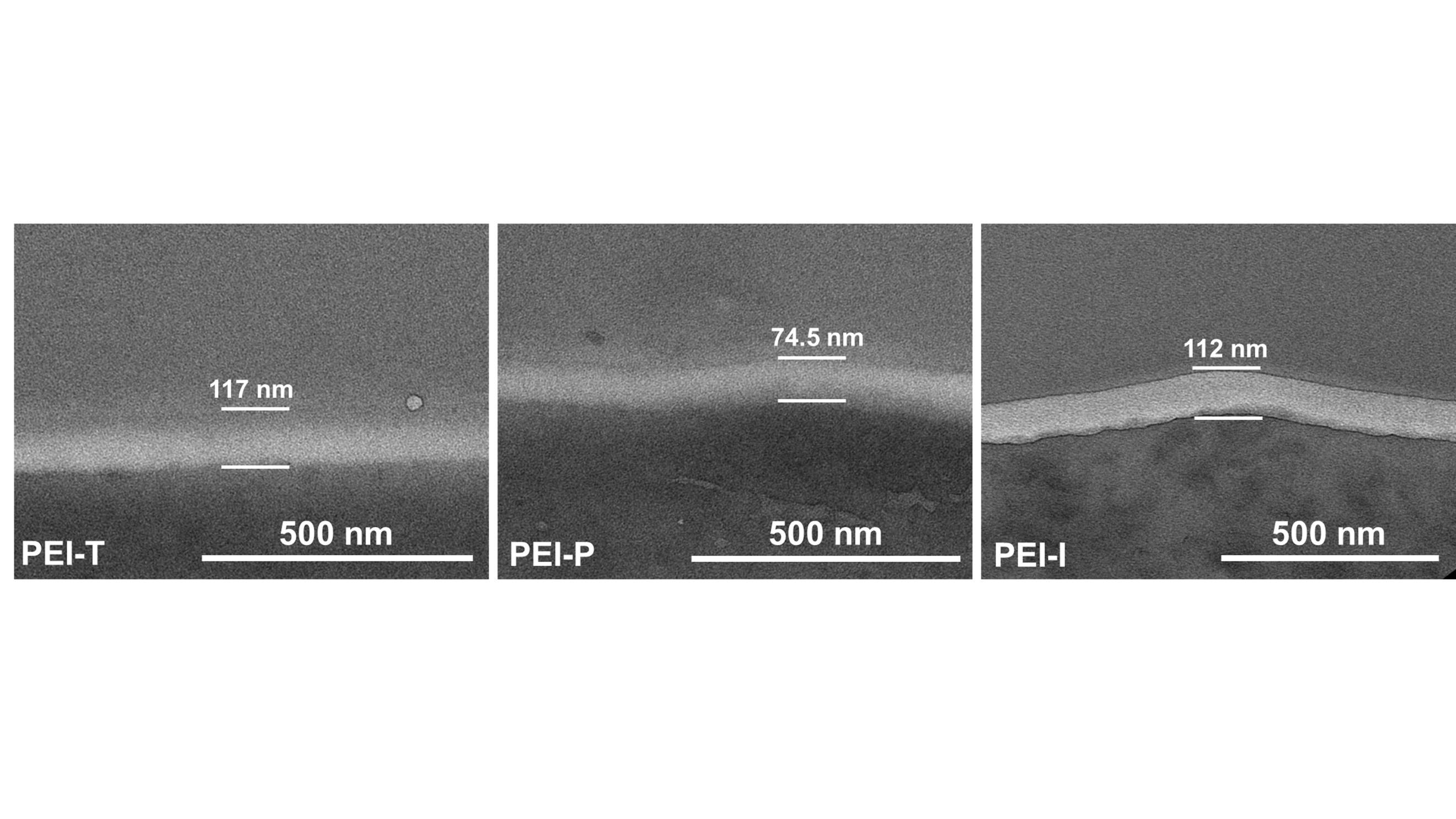


**Figure S7.** Cross-sectioned TEM image of a slice of PEI-T, PEI-P, and PEI-I membrane.


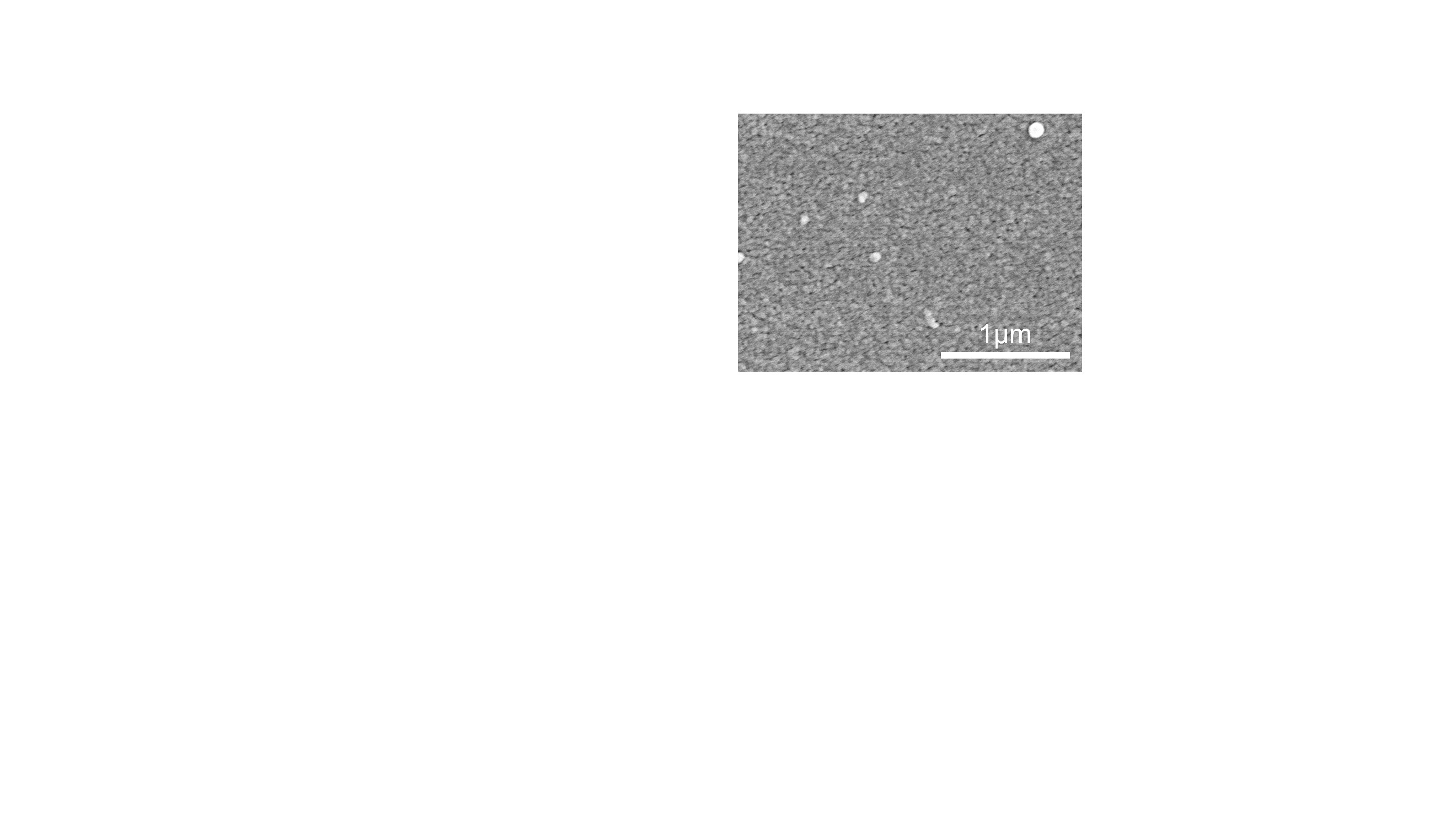


**Figure S8.** Top view SEM image of PSF membrane.


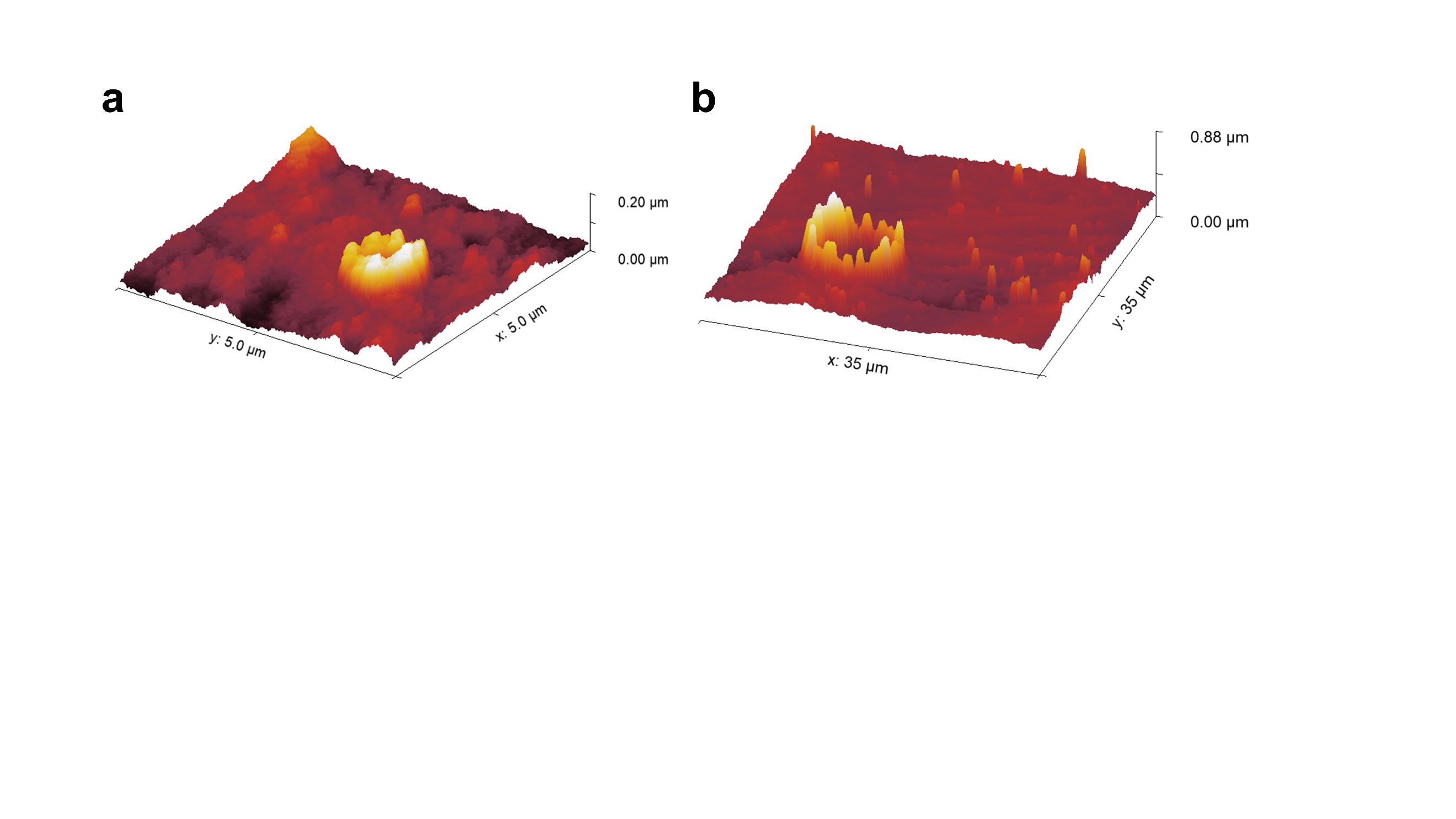


**Figure S9.** (a) AFM topography image of the PEI-P membrane. The bright yellow region corresponds to the formed coffee ring protrusion structure. (b) AFM topography image of PEI-P membrane (35 μm× 35 μm).


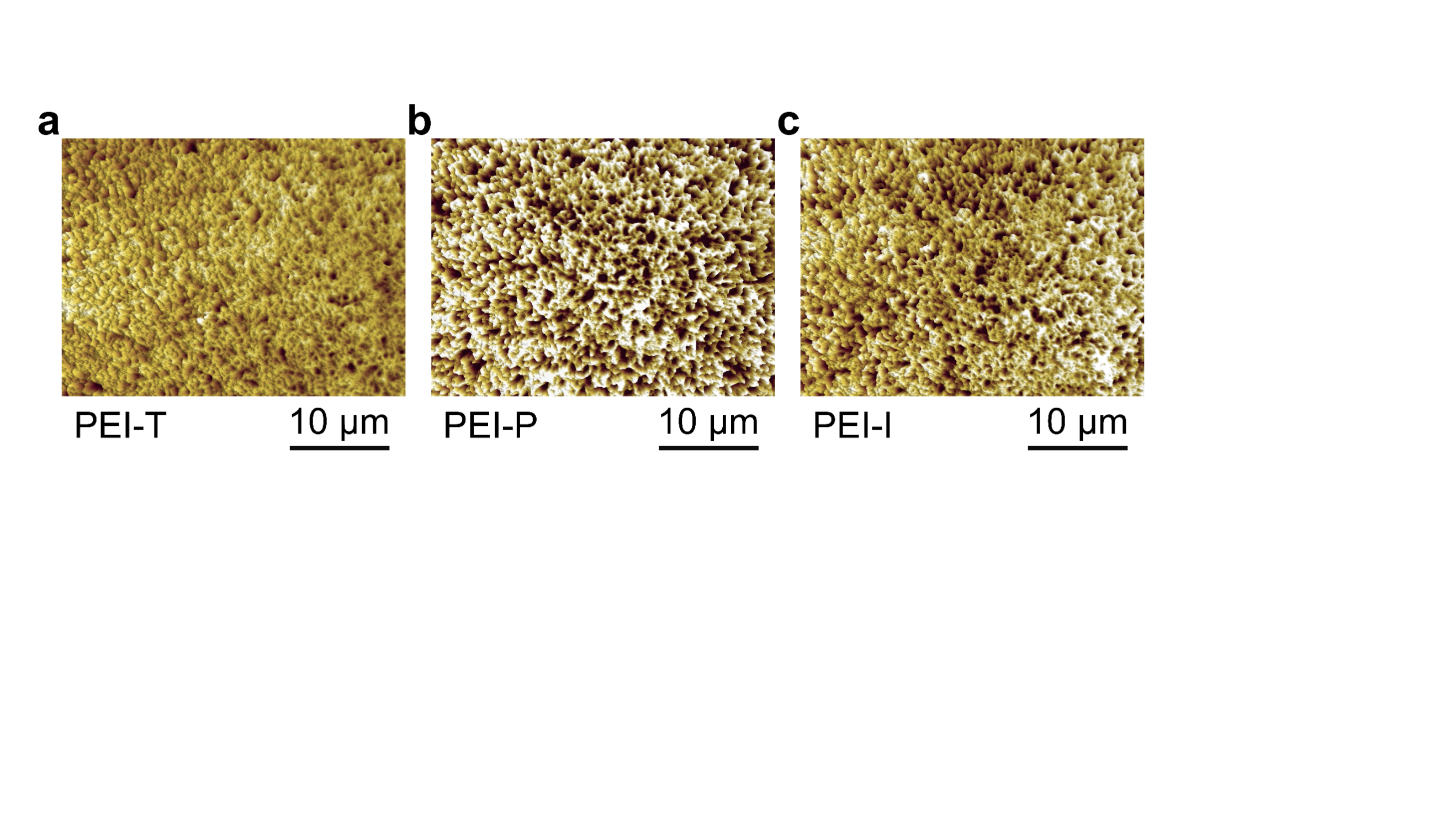


**Figure S10.** Surface profile of (a)PEI-T, (b) PEI-P, (c) PEI-I membranes.


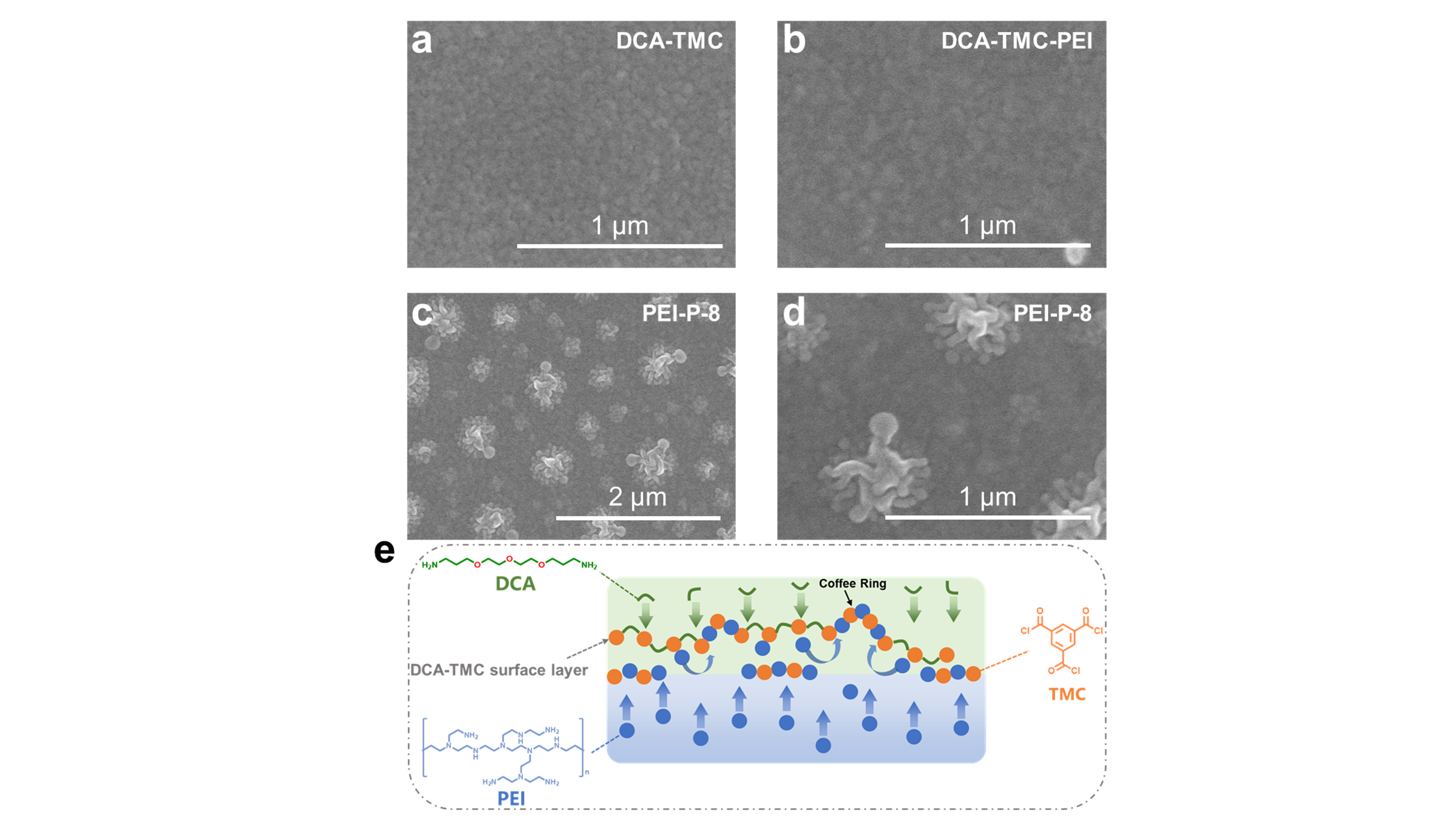


**Figure S11.** Top-view SEM images of (a) DCA-TMC and (b) DCA-TMC-PEI membranes. (c), (d) Top-view SEM images of PEI-P-8 membrane at different magnifications. (e) Schematic diagram of the formation of the coffee ring structure.


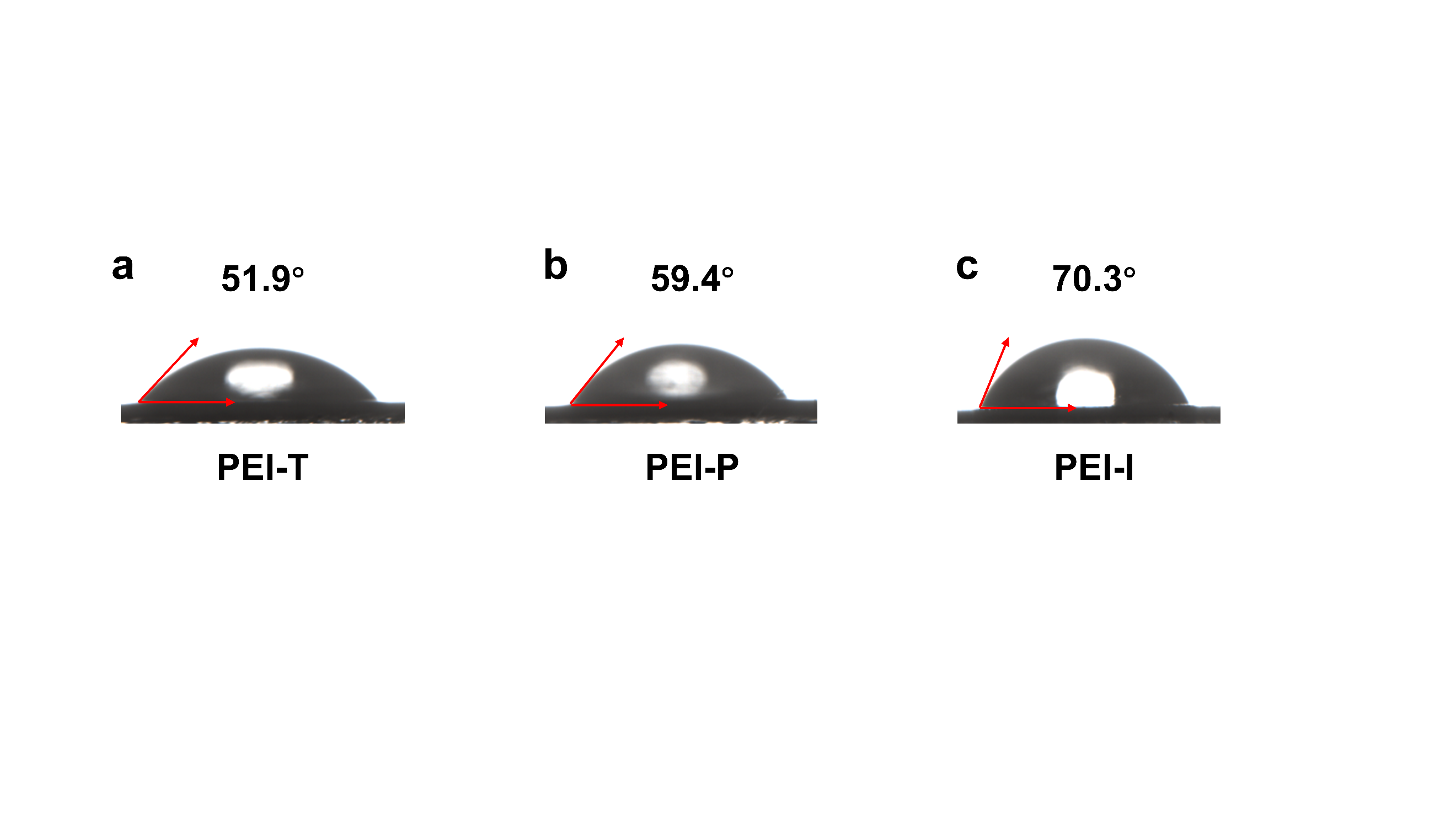


**Figure S12.** Digital photos of water contact angle of (a) PEI-T, (b) PEI-P, (c) PEI-I membranes.


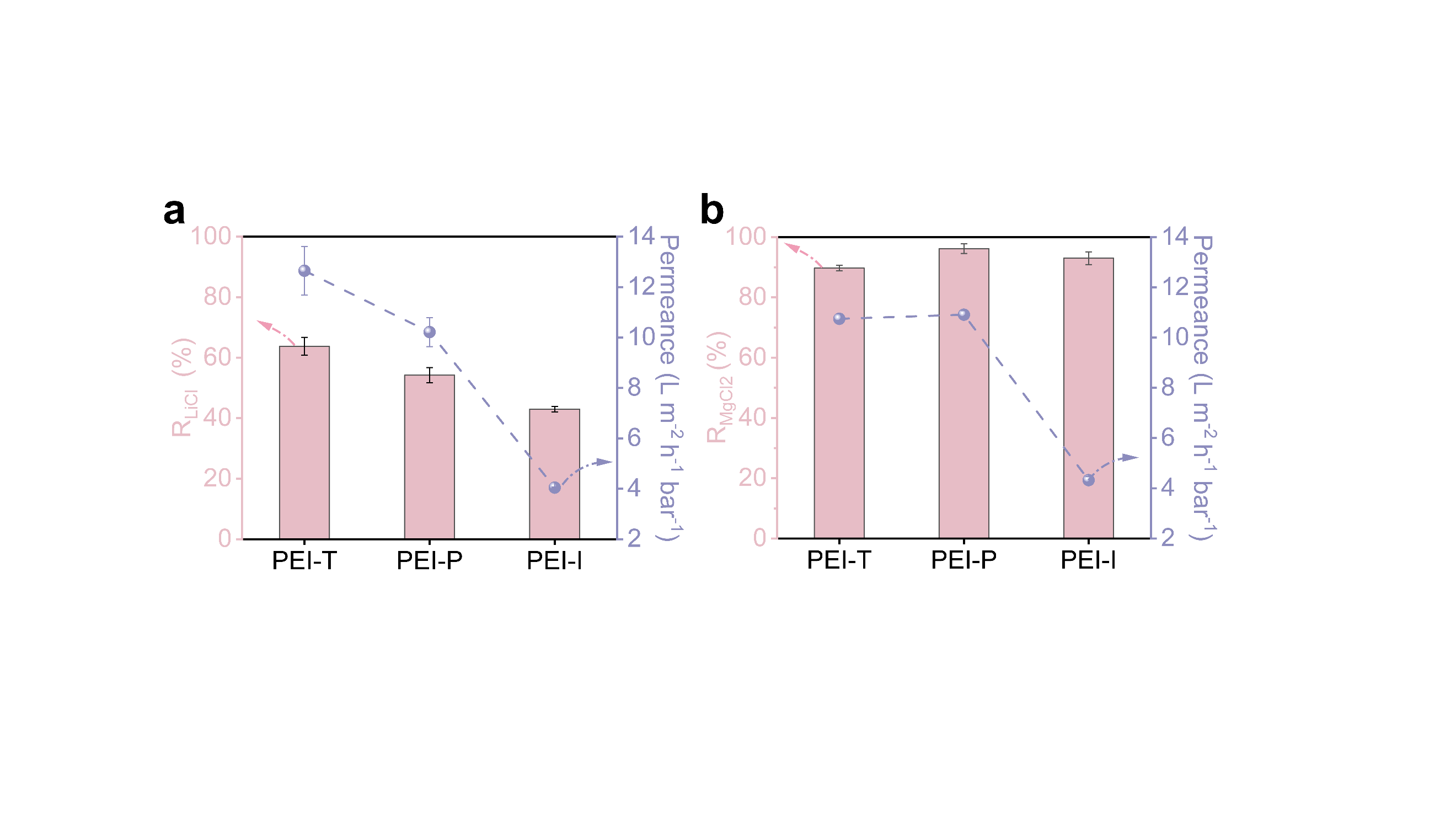


**Figure S13.** The separation performance of PEI-T, PEI-P, and PEI-I membranes was evaluated using 1 g L^-1^ aqueous solutions of (a) LiCl and (b) MgCl_2_ at 6 bar. Error bars in **a**, **b** represent the SD (n = 3) and data are presented as mean values ± SD.


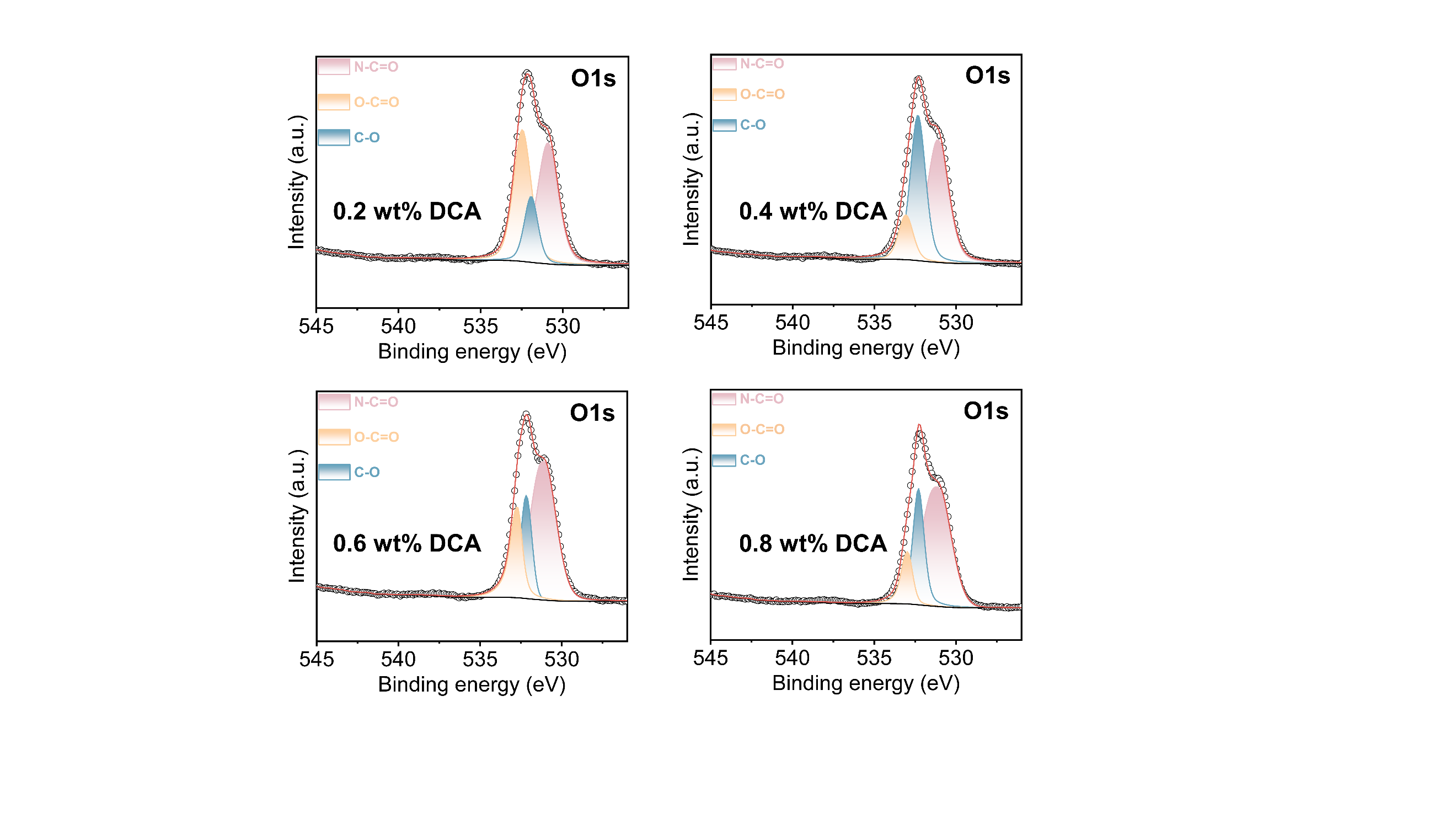


**Figure S14.** High-resolution O1s XPS spectra of PEI-P membranes prepared using different concentrations of DCA monomer.


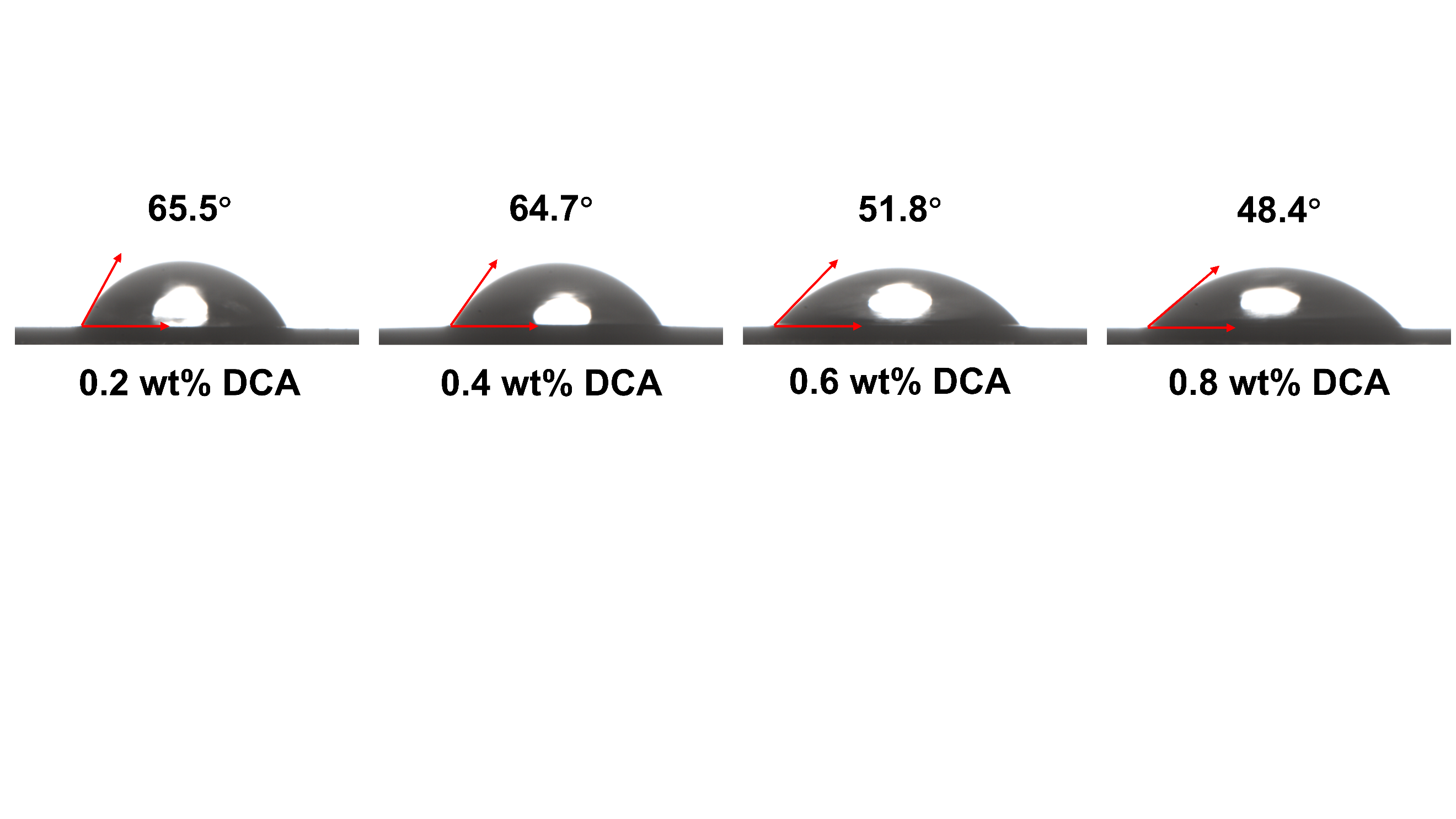


**Figure S15.** Digital photos of water contact angle of PEI-P membranes prepared using different concentrations of DCA monomer.


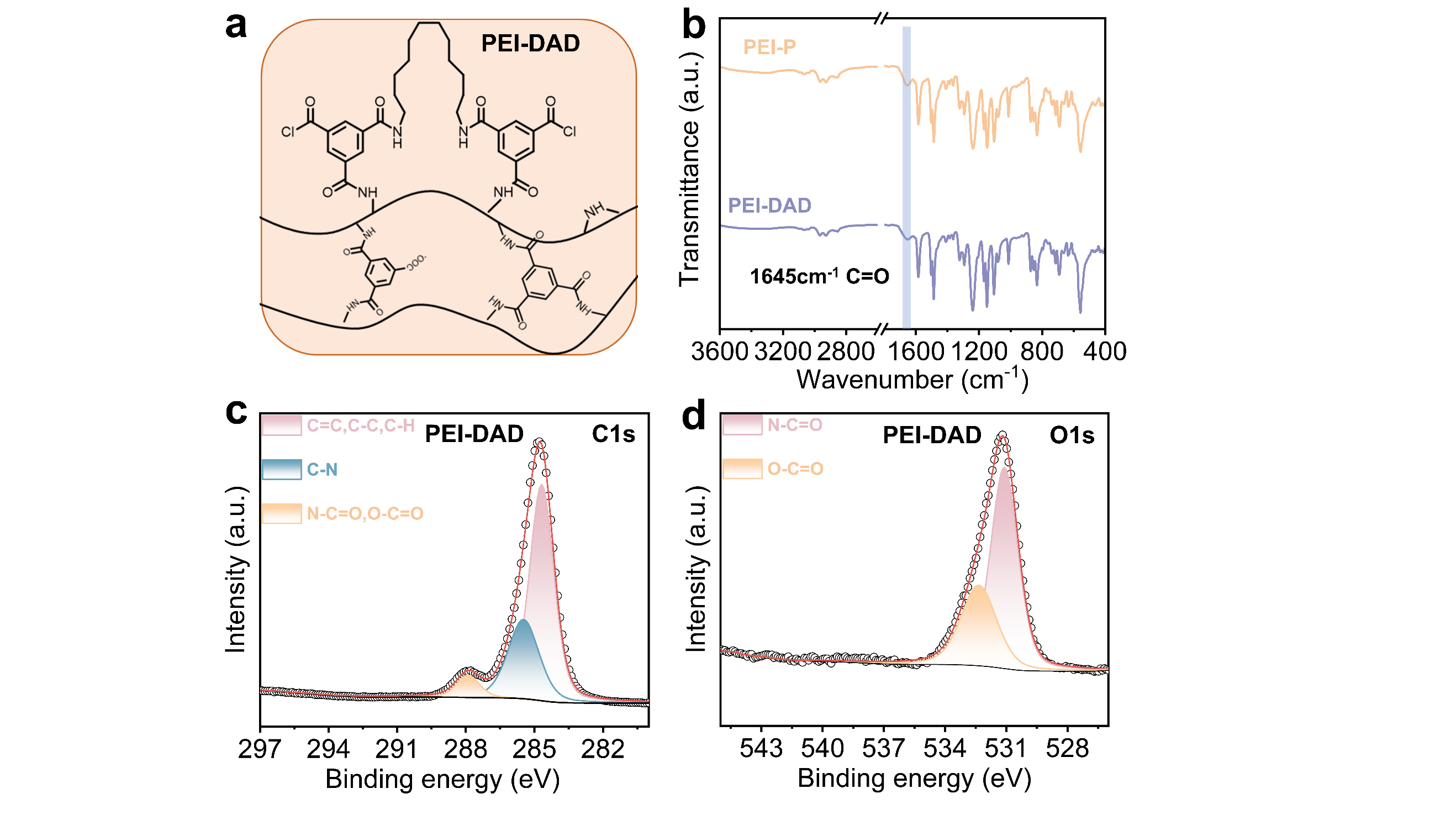


**Figure S16.** (a) Schematic structure of PEI-DAD membrane. (b) ATR-FTIR spectra of PEI-P and PEI-DAD membranes. (c) High-resolution C1s XPS spectra of PEI-DAD membranes. (d) High-resolution O1s XPS spectra of PEI-DAD membranes.

**
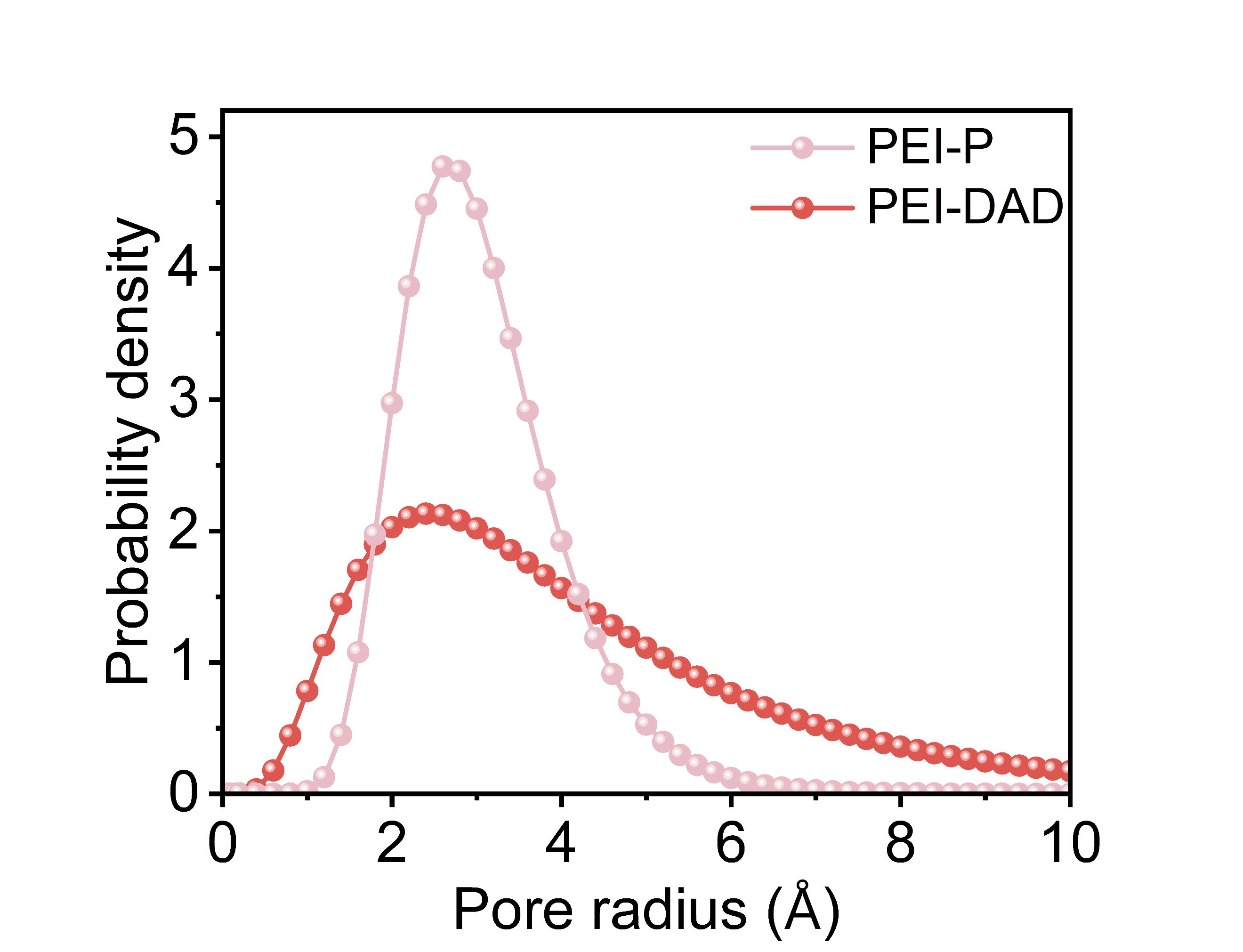
**

**Figure S17.** Pore size distributions of PEI-P, and PEI-DAD membranes.

**
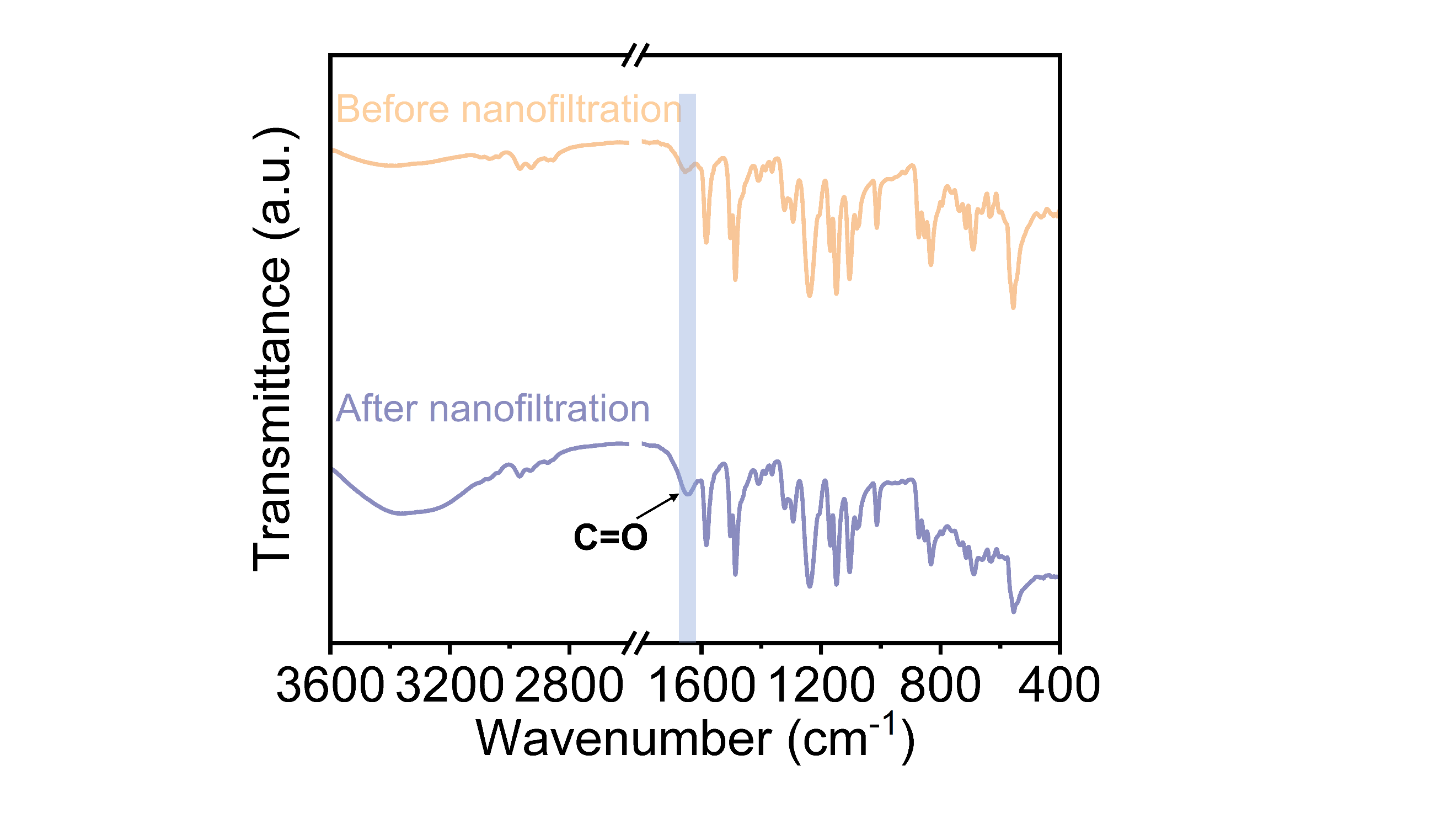
**

**Figure S18.** The FTIR spectra of PEI-P membranes before and after nanofiltration testing.

**Table S1.** Element contents of PEI-T, PEI-P, PEI-I, and PEI-DAD membranes measured by XPS.

| Membranes | C (%) | N (%) | O (%) |
| --- | --- | --- | --- |
| PEI-T | 70.1 | 15.3 | 14.6 |
| PEI-P | 75.0 | 7.8 | 17.13 |
| PEI-I | 71.9 | 8.8 | 19.3 |
| PEI-DAD | 81.3 | 8.5 | 10.2 |

**Table S2.** The analysis results of the XPS core-level C1s spectra of the PEI-T, PEI-P, PEI-I, and PEI-DAD membranes.

| Membranes | Energy (eV) | Species | (at%) |
| --- | --- | --- | --- |
| PEI-T | 284.7 | C=C, C-C, C-H | 53.5 |
|  | 285.7 | C-N | 36.3 |
|  | 287.6 | C=O | 10.2 |
| PEI-P | 284.7 | C=C, C-C, C-H | 59.5 |
|  | 285.6 | C-N | 14 |
|  | 286.3 | C-O | 18.9 |
|  | 287.8 | C=O | 7.6 |
| PEI-I | 284.8 | C=C, C-C, C-H | 57.7 |
|  | 258.7 | C-N | 11.4 |
|  | 286.3 | C-O | 21.7 |
|  | 287.7 | C=O | 9.2 |
| PEI-DAD | 284.7 | C=C, C-C, C-H | 64 |
|  | 285.5 | C-N | 29.7 |
|  | 287.9 | C=O | 6.3 |

**Table S3.** The analysis results of the XPS core-level O1s spectra of the PEI-T, PEI-P, PEI-I, and PEI-DAD membranes.

| Membranes | Energy (eV) | Species | (at%) |
| --- | --- | --- | --- |
| PEI-T | 530.8 | N-C=O | 81.0 |
|  | 532.3 | O-C=O | 19.0 |
| PEI-P | 531.2 | N-C=O | 54.4 |
|  | 532.3 | C-O | 15.7 |
|  | 532.8 | O-C=O | 30.0 |
| PEI-I | 531.1 | N-C=O | 47.4 |
|  | 532.3 | C-O | 40.6 |
|  | 533.1 | O-C=O | 12.0 |
| PEI-DAD | 531.1 | N-C=O | 65.9 |
|  | 532.4 | O-C=O | 43.1 |

**Table S4.** The analysis results of the XPS core-level O1s spectra of PEI-P membranes prepared using different concentrations of DCA monomer.

| Membranes | Energy (eV) | Species | (at%) |
| --- | --- | --- | --- |
| 0.2 wt% DCA | 530.9 | N-C=O | 43.7 |
|  | 531.9 | C-O | 15.7 |
|  | 532.5 | O-C=O | 40.6 |
| 0.4 wt% DCA | 531.2 | N-C=O | 54.4 |
|  | 532.3 | C-O | 15.7 |
|  | 532.8 | O-C=O | 30.0 |
| 0.6 wt% DCA | 531.1 | N-C=O | 59.2 |
|  | 532.2 | C-O | 18.5 |
|  | 532.7 | O-C=O | 22.3 |
| 0.8 wt% DCA | 531.1 | N-C=O | 60.4 |
|  | 532.3 | C-O | 27.2 |
|  | 533.0 | O-C=O | 12.4 |

**Table S5.** Comparison of separation performance of PEI-P with other Mg^2+^/Li^+^ separation membranes.

| Membrane | C_feed_ (ppm) | Mg^2+^/Li^+^ ratio in feed | Permeance  (LMH/bar) | S_Li,Mg_ | Ref |
| --- | --- | --- | --- | --- | --- |
| PEI-TMC | 2000 | 20 | 5.2 | 20 | ^[1]^ |
| PES/CQDsNH_2_/TMC | 2000 | 20 | 12 | 14.4 | ^[2]^ |
| PIL-TMC | 2000 | 20 | 11 | 10 | ^[3]^ |
| RIP-0.250 membrane | 2000 | 20 | 0.8 | 9.2 | ^[4]^ |
| PIP-MWCNTs | 2000 | 21.4 | 8.5 | 16.4 | ^[5]^ |
| PHF-doped TFC | 2000 | 21.4 | 6.7 | 13.1 | ^[6]^ |
| PEI@15C5 | 2000 | 20 | 8 | 14 | ^[7]^ |
| PEI-LDH/GA/PAN | 1000 | 10 | 4 | 18 | ^[8]^ |
| PA/PE-TFC-IPA | 2000 | 20 | 4.8 | 18 | ^[9]^ |
| [MimAP][Tf_2_N]-PA | 2000 | 20 | 4.7 | 8.1 | ^[10]^ |
| BPEI/TMC/EDTA | 2500 | 24 | 0.6 | 9.2 | ^[11]^ |
| PEI-TMC-QBPD | 2000 | 50 | 13.6 | 5.9 | ^[12]^ |
| PEI-TMC-HMTAB | 2000 | 50 | 16.3 | 10.2 | ^[13]^ |
| PEI-TMC-QEDTP | 2000 | 50 | 18.8 | 15.6 | ^[14]^ |
| PEI/TMC/CNCCOOH | 2000 | 60 | 3.4 | 5.8 | ^[15]^ |
| (PES-GO)/PEI/TMC | 2000 | 20 | 11.2 | 16.1 | ^[16]^ |
| Cu−MPD membrane | 2000 | 23 | 16.2 | 8 | ^[17]^ |
| PBI_12-25K | 2000 | 10 | 2.8 | 15.2 | ^[18]^ |
| IP membrane | 2000 | 20 | 0.43 | 3.25 | ^[19]^ |
| SERS-0.50 | 2000 | 20 | 2.26 | 7.68 |  |
| SIP-0.15 | 2000 | 20 | 1.3 | 15.38 |  |
| NF-90 | 2000 | 20 | / | 2.1 | ^[20]^ |
| PEI/Cyclen-TMC | 2000 | 20 | 14 | 8.7 | ^[21]^ |
| PEI/PIP-TMC | 2000 | 30 | 10.6 | 18.26 | ^[22]^ |
| **This work** | **2000** | **20** | **7.98** | **23.3** |  |

References

[1] P. Xu, W. Wang, X. Qian, H. Wang, C. Guo, N. Li, Z. Xu, K. Teng, Z. Wang, *Desalination.* **2019**, *449*, 57.

[2] C. Guo, X. Qian, F. Tian, N. Li, W. Wang, Z. Xu, S. Zhang, *Chem. Eng. J.* **2021**, *404*, 127144.

[3] Y. Ni, H. Peng, Q. Zhao, *Adv. Mater. Interfaces.* **2022**, *9*, 2201797.

[4] Y. Li, S. Wang, W. Wu, H. Yu, R. Che, G. Kang, Y. Cao, *J. Membr. Sci.* **2022**, *659*, 120809.

[5] H.-Z. Zhang, Z.-L. Xu, H. Ding, Y.-J. Tang, *Desalination.* **2017**, *420*, 158.

[6] Q. Shen, S.-J. Xu, Z.-L. Xu, H.-Z. Zhang, Z.-Q. Dong, J. Appl. Polym. Sci. 2019, 136, 48029.

[7] H. Li, Y. Wang, T. Li, X.-K. Ren, J. Wang, Z. Wang, S. Zhao, *Chem. Eng. J.* **2022**, *438*, 135658.

[8] H. Ni, N. Wang, Y. Yang, M. Shen, Q.-F. An, *Desalination.* **2023**, *548*, 116256.

[9] Q. Wang, Y. Dong, J. Ma, H. Wang, X. Xue, C. Bai, M. Lin, L. Luo, C. Gao, L. Xue, *Desalination.* **2023**, *553*, 116463.

[10] H. Wu, Y. Lin, W. Feng, T. Liu, L. Wang, H. Yao, X. Wang, *J. Membr. Sci.* **2020**, *603*, 117997.

[11] W. Li, C. Shi, A. Zhou, X. He, Y. Sun, J. Zhang, *Sep. Purif. Technol.* **2017**, *186*, 233.

[12] Y. Feng, H. Peng, Q. Zhao, *Sep. Purif. Technol.* **2022**, *280*, 119848.

[13] H. Luo, H. Peng, Q. Zhao, *Appl. Surf. Sci.* **2022**, *579*, 152161.

[14] Y. Xu, H. Peng, H. Luo, Q. Zhang, Z. Liu, Q. Zhao, *Desalination.* **2022**, *526*, 115519.

[15] C. Guo, N. Li, X. Qian, J. Shi, M. Jing, K. Teng, Z. Xu, *Sep. Purif. Technol.* **2020**, *230*, 115567.

[16] P. Xu, J. Hong, X. Qian, Z. Xu, H. Xia, Q.-Q. Ni, *Desalination.* **2020**, *488*, 114522.

[17] L. Wang, D. Rehman, P.-F. Sun, A. Deshmukh, L. Zhang, Q. Han, Z. Yang, Z. Wang, H.-D. Park, J. H. Lienhard, C. Y. Tang, *ACS Appl. Mater. Interfaces.* **2021**, *13*, 16906.

[18] O. Setiawan, Y.-H. Huang, Z. G. Abdi, W.-S. Hung, T.-S. Chung, *J. Membr. Sci.* **2023**, *668*, 121269.

[19] Y. Li, S. Wang, H. Li, D. Liu, Y. Jin, G. Kang, Y. Cao, *Sep. Purif. Technol.* **2023**, *306*, 122552.

[20] X. Li, C. Zhang, S. Zhang, J. Li, B. He, Z. Cui, *Desalination.* **2015**, *369*, 26.

[21] T. Li, X. Zhang, Y. Zhang, J. Wang, Z. Wang, S. Zhao, *Desalination.* **2023**, *556*, 116575.

[22] C. Guo, Y. Qian, P. Liu, Q. Zhang, X. Zeng, Z. Xu, S. Zhang, N. Li, X. Qian, F. Yu, *ACS Appl. Mater. Interfaces.* **2023**, *15*, 4814.
